# Supplementary material for: Determinants of clinician and patient to prescription of antimicrobials: Case of Mulanje, Southern Malawi
Source: PLOS Glob Public Health. 2022 Nov 16;2(11):e0001274. doi: 10.1371/journal.pgph.0001274 (PMC10022363; doi:10.1371/journal.pgph.0001274)
Supplement: S1 Text — (DOCX) [file pgph.0001274.s002.docx]

**1. Apependix:1, In-depth interview with clinician number 1,** **on determinants of antimicrobial prescriptions in Mulanje District, Malawi.**

Q: Good morning, ah, my fellow clinician

R: Good morning

Q: We are going to have an interview. You are free not to mention your name in the interview. Whatever we will be discussed. We will be kept in a secret place.

R: Okay, thanks.

Q: You are free to terminate the interview anytime that you want to terminate it and when you feel that the questions are not to your relevant to you.

R: Okay

Q: What is your role at this hospital as a clinician?

R: Medical Assistant

Q: Okay. Where do you conduct the majority of your work?

R: Adult outpatient admission department

Q: Do you prescribe antimicrobials?

R: Yes

Q: Which one do you prescribe most; the anti-malaria and the anti-biotic?

R: Cotrimoxazole, Amoxilline and LA

Q: Why do you think you do prescribe these antimicrobials frequently?

R: Clients that come each and every day, the complaints that they have it’s due to cough and some chest pains which the prescription they need is the anti-biotics. The first line anti-biotics like Cotrimoxazole and amoxicillin.

Q: on average per day how many times do you prescribe antimicrobials?

R: any times; 3 to 4 times

Q: okay. Can you share me patient’s factors that influence antimicrobials prescription? So what I mean when a patient has come to the hospital, okay?

R: umh

Q: what patient factors do influence you to prescribe antimicrobials?

R: the complaints that they have, like that; complaints.

Q: Complaints?

R: yeah.

Q: okay. Any other factor?

R: according to their health profile, maybe before they come to the hospital that day maybe a week before they also came with the same complaint so maybe the clinician that time prescribed Cotrimoxazole so we sort of prescribe another antibiotics like amoxicillin.

Q: okay

R: yeah

Q: complaints health profile previous history?

R: umh

Q: any other factor?

R: no,

Q: You don’t have another factor?

R: yeah.

Q: when do they start prescribing antimicrobials?

R: 2013

Q: okay 2013. So you have mentioned patients factors that influence antimicrobials prescription; patient’s complaints. Health profile, okay previous history, okay?

R: yeah

Q: and you have also mentioned that you started in 2013

R: Yes

Q: prescribing antimicrobials

R: yeah

Q: Okay. What problems do you face during this period when you started prescribing antimicrobials?

R: okay, some clients complain that when you prescribe some antibiotics like Cotrimoxazole maybe before they finish the dosage, they always come that there is no change about the antibiotic which you prescribed that day. So some issues that they complain that there is no change on the prescription…yeah.

Q: Okay, without finishing their prescribed dosage?

R: yes

Q: okay. Any other problem?

R: okay the other thing is that there are some clients or some patients that came to the hospital yet they are not sick.

They just came to the hospital maybe just because they have come to see a patient in the ward so they prefer to be prescribed some drugs. Or we just we.., or sometimes I heard some guardians like in the afternoon, they always come to the OPD to collect drugs that when going home they should have at least enough medication yet they are not sick.

Q: okay, any other problem?

R: The other is when we prescribe those antibiotics, I believe they don’t take medicines at all.

Q: okay, any other?

R: you may feel that they come to the Outpatient Department and complain that there was a certain day I came and they prescribed me some antibiotics or some antimicrobial yet in the file you see there is nothing in the passport or when you go through the passport that there is a lot of antibiotics that the patient is on. But if you ask them sometimes they that I did not take the medication I thought it’s not helpful though I need some extra drugs.

Q: okay

R: yeah

Q: there is another thing that you want to add on this?

R: no.

Q: okay. So you have mentioned not finishing their doses when they have prescribed antibiotic.

R: yes.

Q: aaah, sometimes they come to the hospital where they are not sick they just want to get medication. Sometimes they come to see sick patients, okay?

R: yeah

Q: like seeing patients they will come to the OPD to get medication.

R: Yeah

Q: You have also mentioned that guardians do come to the OPD so that to get medication so that when they go home they should go with enough though they are not sick

R: yes

Q: sometimes aah, you also said aah, they come to the hospital and found that in the health profile there is a lot of antibiotics but when you ask them they say that they feel like the drug was not necessary.

R: Yeah

Q: okay, can we proceed?

R: yeah

Q: explain to me one thought regarding to patient factors and beliefs ant microbial? What do your patients believe about anti-malaria and antibiotics?

R: okay about the antibiotics.

Q: Their belief what do they believe; antimicrobials and both antimalarial and antibiotics?

R: Some, most of the clients, they believe that the antibiotics are more help than other drugs and also the antimalarial. You feel that they come and you ordered MRDTs, MRDTs becomes negatives, they push you to write antimalarial yet the MRDTs is negative. They feel that when they get malarial, antimalarial they will be fine yet they are not sick for malaria.

Q:okay

R: Yeah

Q: Any belief?

R: no

Q: That’s all?

R: Yeah

Q: Okay, so you mentioned that patient believe that antibiotic are more helps than other drugs

R: yeah

Q: you have mentioned that when patient comes to the hospital, you test them for MRDTs come up negative they will push you to prescribe antimalarial because they believe that when they get antimalarial they will feel better while they are not sick.

R: yeah

Q: they don’t have malaria

R: yes

Q: okay. In your view how do you describe the attitude of patients when it feels to prescribing antimicrobials? So the patient has come so you have sent FBC, it’s okay

R: yeah

Q: you have seen the MRDTs, is negative. The FBC are normal so say go home and take a lot of fluids you will be fine, how is your patient react to you? What’s they attitude?

R: Sometimes they, they change the complaints that they have. Once you have told that your FBC is okay, your MRDTs is negative go home and do some exercise, drink more fluids then they start having another complaints yet the first time they come they did not mention that complaints. I think they do that like after you prescribe you told those advice maybe you may add another drug like antimicrobials like antimalarial or antibiotics.

Q: okay

R: yeah

Q: any other attitude when you have refused to give medication to your patient?

R: they get angry

Q: they get angry. Any other?

R: I think that’s all

Q: so the attitude you have mentioned that they change their complaints.

R: umh

Q: So they shift to another complaint so that you should prescribe antimicrobial and they get more angry. Okay.

Q: what communication skills are needed when you are prescribing antimicrobials?

R: what?

Q: communication skills; what communication skills are needed when you are prescribing antimicrobials?

R: okay, you need to tell the client or the patient what the results are like you ordered MRDTs, FBC or what then when the results are in you need to, you always need to share the results to our clients

Q: okay?

R: yeah. If the MRDTs is positive we need to tell them that your MRDTs is positive that means you have malaria and we are giving this, you are going to get LA at the pharmacy you need to…and also when they are negative, we also need to tell that your MRDTs is negative and that means you don’t have malaria or the FBC results you need to share to tell them that when the FBC is having the HB of low WBCs, platelets, we need to tell that they should know their results before going home.

Q: okay

R: yes

Q: Any other communication skill?

R: (silence)

Q: just think when you are at the OPD, when you are at adult, when you are at STI, whatever communication skill do you need when you prescribing antimicrobial.

R: okay, we need to, we also need to tell about the dosage and they need to also to finish the dose. The benefits of finishing the dose and complication that they will have and also the side effects of the drugs that we have prescribed those. Each and every drug it has its own side effects so we need to tell them that if you see this that is this; minor side effects if the drug. If you see this that is the side effects, you need to come back to the hospital.

Q: okay, you mentioned of complications; the complication in terms of the drug or the disease?

R: The complication about the drug.

Q: okay

R: Meaning the side effects

Q: okay. So in terms of communications skills you have mentioned that you need to share the results with the patient.

R; mh

Q: okay

R: umh

Q: they have to know the results, okay?

R: Yeah

Q: you also mentioned that the dose, they have to know the dose; they have to finish the dose, okay?

R: yeah

Q: they have to know the benefits

R: yeah

Q: the drug

R: yeah

Q: you mentioned that?

R: yeah

Q: they have to know the complications

R: yes, so the communication skill you need to, we are supposed to tell them, we need to welcome them and also to tell our full name but sometimes with the long lines we are unable to share because there is informations.

Q: okay

R: yeah

Q: so you mentioned of side effects, welcome them, okay?

R: umh

Q: name your introduction

R: yeah

Q: okay. Thank you can we proceed?

R: yes

Q: okay. How much time do you spend with each patient?

R: (laughs) to be frankly honest, 2 minutes

Q: 2 minutes?

R: 1-2 minutes

Q: why do you think it takes?

R: it takes that time because in adult OPD, we have so many clients. Some of them they came from the Health centre nearby our district, our mother hospital. So and workload there

Q: umh

R: is high; hence, we have sometimes I work alone, sometimes you have 2 Clinicians, at least if we are many that means we have 3 Clinicians. But normally, its 2 Clinicians so to finish up the long line, you cannot spend most, you cannot spend more time with the patient. If you spend more time with a patient, those clients, those patients they also get angry. They start talking rubbish to the Clinician that you are wasting their time.

Q: so you say you spend less with the patient because normally you are few. There are so many patients. How does it affect your profession to prescribe antimicrobial?

R: okay, aah, it affects a lot because we need to have more time with our patient and they should talk more of their complaints but with the complaints that I said that we are few Clinicians, we spend aah not enough time with the patient so the patient do they not share more of their complaints that they have come with on that particular day.

Q: okay.

R: yeah

Q: Can we proceed?

R: umh

Q: okay. Can you describe some of the guidelines that are used during the prescription of antimicrobials both antimalarial and antibiotic by the clinician?

R: Guidelines

Q: umh

R: bluebook or?

Q: Any guidelines that you know that are being used by clinicians?

R: okay, MSTG

Q: Okay, MSTG

R: Clinical management and bluebook

Q: okay, bluebook, clinical management

R: and surgical book

Q: Surgical book. Any, any other you know?

R: umh, I have forgotten the book

R: U-guideline of Malaria.

Q: okay, what is the guideline?

R: ART new guideline

Q: ART guidelines

R: that’s all

Q: okay. so you have mentioned MSTG, what is MSTG?

R: (silence)

Q: So you have mentioned MSTG,

R: yeah

Q:Clinincal Medicine Bluebook that what’s you meant it?

R: umh

Q: The surgical handbook

R: yeah

Q: The malaria guidelines

R: Yes

Q: The ART

R: New guidelines

Q: Okay, thank you

Q: Have you ever heard of bacteria resistance?

R: yes

Q: In your own words what is it?

R: okay. Come again about the question.

Q: okay. Have you ever heard about bacteria resistance?

R: yes

Q: So in your own words what is it?

R: okay. umh, when I get antibiotic like Cotrimoxazole then I did not finish the dose and the other time I came to the hospital they also prescribe the same antibiotic and I did not finish the dose or I finish the dose or I take it I take Cotrimoxazole frequently they become resistant in my body. That means when I am having a certain disease like upper respiratory tract infection then they also prescribe the same Cotrimoxazole, it may become resistant in my body. That means I will not get cured.

Q: So what does it mean when you say resistance?

R: umh

Q: Even in Chichewa you can mention it.

Q: Okay, just phrase it then.

R: when you have taken drugs like amoxicillin but the amoxicillin is not responding to the disease I am suffering from although in the past I would get cured upon taking amoxicilline for that particular disease.

Q: thank you

R: Yeah

Q: So you have defined bacteria resistance what about antimicrobial resistance what is meant by antimicrobial resistance?

R: I think the combination of antibiotic and antimalarial, is that so?

Q: no, I am just asking to you (chuckles)

R: I think that.

Q: So what is it mean by combination of anti-malarial and antibiotic?

R: (Stay quiet)

Q: Should we proceed?

R: Yes

Q: Okay. Can you describe factors that lead to antimicrobial resistance both antibiotic and antimalarial?

R: umh?

Q: what do you think are the factors that leads to antimicrobial resistance?

R: Okay

Q: Factors that leads to antimicrobial resistance both anti malaria and antibiotic?

R: Low dosage

Q: Okay

R: Taking drugs frequently

Q: okay. Which dugs?

R: The antimalarial and the antibiotics

Q: okay. What do you mean when you say frequently?

R: okay, meaning each and every time you go to the hospital they prescribe the same antibiotic.

Q: okay, yes.

R: That’s all that I can remember

Q: whose responsibility is to resolve the problem?

R: umh

Q: Whose responsibility is to resolve this problem; antimicrobial resistance?

R: the one who is sick.

Q: so the patient, they are responsible to resolve antimicrobial resistance?

R: Yes

Q: Why?

R: According to the first question, the above question that you have asked me.

Q:umh, okay. on the factors?

R: what?

Q: On the factors?

R: umh

Q: okay, thank you. Do you have anything to add?

R: No

Q: NO, okay. Your recording will be kept confidential. We wish a nice day.

R: Thank you

Q: Thank you for participating in this study

R: Thank you

Q: As I have already said I am doing this study, I am a student at College of Medicine.

R: Yeah

Q: Thank you!

R: Thank you!

END OF INTERVIEW
