# Supplementary material for: Determinants of clinician and patient to prescription of antimicrobials: Case of Mulanje, Southern Malawi
Source: PLOS Glob Public Health. 2022 Nov 16;2(11):e0001274. doi: 10.1371/journal.pgph.0001274 (PMC10022363; doi:10.1371/journal.pgph.0001274)
Supplement: S2 Text — (DOCX) [file pgph.0001274.s003.docx]

**2. Appendix:2, In-depth interview with clinician with number 2, on the determinants of antimicrobial prescriptions in Mulanje District, Malawi.**

Q: Good afternoon, sir!

R: Good afternoon!

Q: I am Maurice Chalusa, Clinical Officer of Mulanje District Hospital. I am also a student at College of Medicine doing Master of Science and Health Sciences Antimicrobial Stewardship. I am doing a study called ‘Determinants of decision between a Clinician and a patient to prescribe Antimicrobial a Clinician Respective.’ Aaah, my interview will take around 20-40 minutes. They are questions, you are free not to mention your name. You are to terminate you think or when you believe that we have violated your rights. Ah the recording of this study will be kept secret. They will not be accessible to anyone else. The results will be shared to you after the study. Thank you.

Q: Can we proceed?

R: Yes

**Q: Okay. aah, what is your role at this hospital?**

R: I am a Clinician.

**Q: Where do you conduct majority of your work?**

R: Yeah, most of the time, most of the times I see more patients in the ward especially Pediatric ward.

**Q: Okay. Do you prescribe antimicrobials?**

R: Yes, I do prescribe antimicrobials.

**Q: okay. aah which one do you prescribe most between antibiotics and antimalarial?**

R: Antibiotics

Q: Antibiotics

R: Yes

**Q: Why do you think you prescribe antibiotics than anti malaria?**

R: I see more patients, aah, with a probably with respiratory infections other than the malaria cases.

Q: okay. Examples of antibiotic that you prescribe mostly?

R: Amoxicillin

Q: okay

R: yes

Q: any other example?

R: Yes, Cipro

Q: Okay, any other example?

R: yes, co-Trimoxazole.

Q: Okay. in terms of antimalarial?

R: in terms of antimalarial, of course we have got Artesunate but I prescribe also LA.

**Q: Okay. How many times do you prescribe per day antimicrobials?**

R: Times a day?

Q: umh, how many times a day?

R: Aah, 3 times a day.

**Q: 3 times a day. Okay, which one are the most frequently prescribed between antimalarial and antibiotic?**

R: Antibiotic especially amoxicillin.

**Q: Okay. Can you share with me what you know about patient factors that influence antimicrobial prescription? What do you think are the factors that influence antimicrobial prescription; patient factors?**

R: Come again.

**Q: Can you share with me patient factors that influence antimicrobial prescription; patient factors that influence you to prescribe antimalarial or antibiotics?**

R: Alright, aah, for example, when the patients with fever, cough, when the shortness of breath, yeah, I think check MRDTs is negative so what you do is you prescribe the antibiotic.

**Q: Any more patient factors?**

R: aah, yes, if the patient is not responding on antimalarial that is already diagnosed malaria patient and that patient is not responding on the antimalarial so you try, its just a matter of try, you try antimicrobial and see whether it will work. That’s could also be another factor.

**Q: Any other factor that can, that got patient influence to prescribe antibiotic or antimalarial?**

R: When its young or adult?

Q: Yes

R: Sometimes some patients would ask you to, would say if I take amoxicillin I respond quite well to my cough, what to do to my condition. You think sometimes patients may also influence somebody to give antibiotics.

Q: Any more factors? You can mention as much as possible.

R: Sometimes, its just a routine. People just think just a matter of thinking that whichever conditions maybe if we give antibiotic that condition will actually respond to the antibiotic.

Q: Okay?

R: Yeah.

Q: Have you exhausted or there are more?

R: aah, I think for the time being let me stop there.

Q: So you have mentioned of cough…

R: Yes

Q: Fever

R: Yes

Q: Shortness of breath

R: Yes

Q: aah, MRDTs when is negative you prescribe antibiotic

: Yes

Q: A patient is not responding to antimalarial

R: Yes

Q: You prescribe antibiotic

R: Yes

Q: You have also mentioned a patient may ask you to give antibiotic. May tell you that okay, if I get amoxicillin I feel better.

R: Yes

Q: Sometimes it’s just a routine to prescribe antibiotic.

R: Yes, I have seen people doing that.

Q: Okay, thank you. Can I proceed?

R: Yes, proceed.

**Q: When did you start prescribing antimicrobials?**

R: aaah as soon as I graduated from college that was in 2014, I started prescribing antimicrobials.

**Q: Okay. Thank you. What are the problem have you faced during this time you started prescribing antimicrobials?**

R: Yeah, some antimicrobials doesn’t work, you prescribe one give it to the patient, the patient doesn’t respond so you go to the other one, you find patient doesn’t respond you go to the other one so there is frequent prescription of antibiotics. When he doesn’t respond to that antibiotic you go to the other antibiotic, he doesn’t respond you go to the other one. So that‘s one of the challenges we have faced so far.

Q: okay, any challenge?

R: Not all antibiotics works.

Q: umh

R: yeah, I don’t know whether I can talk about on the patient side

Q: You can

R: Because some patients do not finish the antibiotics, when they take them for just for a short, few days they come to you I am not responding. So if you doesn’t ask them quite well you prescribe another antibiotic when old antibiotic is still with the patient.

Q: Any more problem?

R:umh, no we can proceed.

Q: okay so in terms of problem you have mentioned that some antibiotic not working if person consume.

R: Yes

Q: In the process you also mentioned frequency prescription of antibiotics as a problem

R: Yes

Q: You also mentioned that other patient do not finish their antibiotic.

R: Yes

Q: They will still come to the hospital while they have other antibiotics at home.

R: Yes and I have an example. There was one case

Q: Okay, thank you

**Q: Can you explain to me your thought regarding patient factors and belief about antimicrobials? What do you think are patient belief about antimicrobials?**

R: There are some patients who believe that aah, whenever they are sick the only drug they should take is antibiotics. Whether it is just only cough, whether is abdominal pain but they take antibiotics. They think so.

Q: Okay

R: Yeah

Q: Any belief?

R: yes. The other one could be aah, some patients they have their own antibiotics that they think they are better than other antibiotics so they keep on taking those antibiotics though there is a wide range of antibiotics. For example, Amoxicillin, most of the patients they go for amoxicillin whether you have Ciproflaxicilline, we have co-Trimoxazole but still more patients they prefer Amoxicillin so that’s another, another factor, another challenge, another problem.

Q: Any other problem?

R: let’s proceed.

Q: Okay. So you have mentioned that ahh, when patients are sick they believe that even when they have just cough they should take antibiotic.

R: Yees

**Q: You have also mentioned that patient they have got their own antibiotics.**

R: Yes

Q: aah, they feel better with some antibiotics like amoxicillin they will continue to take antibiotic. So where do these patient take the Amoxicillin?

R: Some of course they buy.

Q: They buy

R: umh, some it’s we clinicians who prescribe these antibiotics to them.

Q: So how, how do they even approach you?

R: Some are our friends, some are our relatives so we just give them the antibiotics considering that when you deny them, when you say no they will think (stammers), you may create enmity between them.

Q: Okay

R: Yeah

**Q: Since you have started prescribing antimicrobials, what are the challenges?**

R: (Silence) the challenges could be dosing of antibiotics.

**Q: What do you mean when you say dosing?**

R: Dosing?

Q: Umh

R: How…

**Q:How is dosing a challenge?**

R: A challenge its like still its difficult to know aahh the other dosing of other antibiotics. For example, I would say some patients do not take the right dosage of antibiotics, maybe I can put it that way. Aahm…

Q:Okay

R: So it’s a challenge. The other one could be as I have said some antibiotics do not work on the conditions they used to work previously. Okay

Q:Umh

R: Yeah. Previously the antibiotic was working but now we give to patients with same condition doesn’t work. Aaaum, the other one is aahum…I think that could be those.

Q: okay, so you have mentioned challenges that dosing; other patients do not take the right doses. Aah you have also antibiotic not working to certain conditions previous used to work.

R: yeah, yes and the other thing knowing the side effects of the antibiotics, it’s a challenge.

**Q: To whom?**

R: To…even the Clinicians or even the patients themselves. But we just prescribe those antibiotics, on the part of clinicians, without explaining the side effects for those antibiotics.

Q: Okay

R: So I think it’s a challenge also.

**Q: Okay. Any more challenges**

R: aah, the other challenge is for how long should the patient take the antibiotics? Because now if you ask me how long should the patient take Amoxicillin? I would say 5 days but does it mean that the Amoxicillin should be taken 5 days only? To some of the Clinicians I think we have, the knowledge that we have, it’s you know, I would say how long should the antibiotics be taken when we also have a knowledge deficit that its usual now 5 days antibiotic, 5 days antibiotic. But maybe some of these can be taken for so long. That’s why maybe they don’t work.

Q: okay. So challenge you have mentioned aah, patient not knowing doses, not taking right doses okay?

R:Yeah

Q: aah, antibiotic that used to work on the previous, currently they are not working.

R: Yes

Q: Some of the Clinicians doesn’t know the side effects of antibiotics.

R: Yes.

Q: And even patient as well.

R: Yes

**Q: Some of the Clinicians doesn’t know how long should antibiotics should be taking to each patient.**

R: Yes

Q: And the patient doesn’t know.

R: Yes

Q: That what you have explained.

R: yes

Q: Okay, can we proceed?

R: Yeah, let’s proceed.

**Q: In your view how do you describe the attitude of your patients when you refuse to prescribe antimicrobials?**

R: They are angry.

Q: they are angry.

R: because what they need is antibiotic. If you don’t prescribe them they are not happy.

Q: Okay, they are angry. Any other attitude to you?

R: They feel like you haven’t assisted them.

Q: They feel like you haven’t assist them.

R: umh

Q: Okay. Any other attitude.

R: I think the last thing they go to another Clinician.

Q: okay

R: eeh, for the same antibiotic because what they need is the antibiotic.

Q: so in terms of attitude, you have mentioned that aah, they become angry towards you, they feel you haven’t assisted them and they go to another Clinician.

R: Yes they go to another Clinician.

Q: For the same

R: For the same medical issue.

Q: Okay, can we proceed?

R: Yes

**Q: okay, what communication skills are needed when you are prescribing antimicrobials?**

R: (sniffs, silence) on the communication skills as a Clinician, I have to explain clear on the importance of antibiotics and how the antibiotics should be taken.

**Q: umh, any other communication skills? You ca mention as much as possible.**

R: okay, ummmh. (silence) I think maybe we should…

**Q: That’s the only one?**

R: Yes, I think the matter is to talk to them how they should take the drugs. They should not keep the drugs at home or just take them for few days and then they expect to take them another day. They feel like they are cured. But they need to complete the whole dose of the antibiotics other than leaving half taking half.

Q: okay

R: umh, that’s what I feel maybe that’s all.

Q: okay, so in terms of communication skills you are saying explain the importance of taking antibiotic to your patient. You have also mentioned talking, talk to them how to take the antibiotics.

R: Yes, and the other thing I forgot is also telling them that if they don’t finish antibiotics, they will feel like they are cured but still the infection will resurface.

Q: Okay, thank you.

R: Yes

**Q: Okay, how much time do you spend with your patient?**

R: Time?

Q: umh

R; it depends on the, it depends on the severity of illness.

Q: umh

R: of the patient. As I said I work in the ward and sometimes OPD. But in the ward, it should be about 10 minutes.

Q: Okay

R: When I am reviewing a patient it should be 10 plus minutes but sometimes maybe less than that.

Q: Okay.

R: But on average it should be 10 minutes.

**Q: While others you have got 10 minutes why others you got less time.**

R: Maybe others just think maybe those that have responded, of course you ask them to continue to just continue medication when you are in the ward, yeah. But those that are seriously sick you think of changing the medication. Maybe you have given a different antibiotic or drug then you switch to other drug.

Q: okay

R: eeh

**Q: and how does this time affect antimicrobial prescription? What do you think?**

R: times?

**Q: How this time; the time you spend with the patient, how does it affect antimicrobial prescription?**

R: (silence)…time…yes, aah because there is a time a patient is supposed to take antimicrobial that is according to probably the half-life of the antimicrobial. So I think you take, you would see, umh. A patient can take, aah, antimicrobial maybe not at a recommended, at a recommended time, maybe you just prescribe takes antibiotics does what, maybe that also can affect the microbial.

**Q; okay. Can you describe some of the guidelines used during antimicrobial prescription; antibiotics and antimalarial by Clinicians?**

R: We have of course, MSTG.

Q: What do you mean by MSTG?

R: Malawi Standard Treatment Guidelines

Q: okay

R: 2015

Q: Okay

R: yeah

**Q: another guideline?**

R: there is also the guideline which prescribe antimicrobial.

Q: umh, any guidelines that are used during the prescription of antimicrobials

R: yes, even the HIV guidelines because there are conditions there.

Q: okay, HIV guidelines.

R: there is also second…clinical handbook

Q: what coulour is it?

R: Colour? Blue; bluebook

Q: okay

R: yes, there is also white book pediatric.

Q: White book pediatric.

R: yes.

Q: Any other guideline?

R: aah, there I also ah obstetrics and gynecology. At Queens there are also antibiotics being used in that book.

Q: okay

R: yeah

Q: you can mention as much as possible.

R:okay. Should I continue?

Q; yeah, continue!

R: okay, there is also illustrated pediatric book 4^th^ Edition. There is also, there, there is also antibiotics.

**Q: so you have mentioned Malawi Standard Treatment Guidelines (MSTG)**

R: Yes

Q: HIV guidelines

R: Yes

Q: THE Clinical Bluebook

R: yes

Q: You are studying pediatric.

R: yes

Q: The white book for Peads

R: yes

Q: the O and G, Queen Elizabeth Central hospital handbook

R: yes

Q: When you are studying pediatric

R: yes and I forgot the STI guidelines.

Q: the STI guidelines

R: Yes, there is also antibiotics being used there.

Q: okay, can we proceed?

R: yes.

**Q: Have you ever heard of bacterial resistance?**

R: Yes

**Q: In your own words what is it?**

R: Resistance, aah, in my own opinion, in my own opinion maybe its just aah, some antibiotics not effective onto the condition they used to be treated. Maybe I have to say so.

Q: okay so antibiotics resistance some conditions who not responding to the antibiotic that was previously given

R: yes, being…sensitive eeh

Q; Previous sensitive

R: sensitive previously but now they are not to those antibiotics so I think we would say there is antibiotics resistance.

**Q: okay,, aah, thank you. So what is meant by antimicrobial resistant?**

R: antimicrobial resistance?

Q: eeh

**R: yeah, it’s some microorganism they are not sensitive to the antibiotics. That’s maybe I can define it. Yeah, yeah, yeah.**

Q: What factors leads to antimicrobials resistance?

R: 1 aah, it could be unnecessary prescription of antibiotics.

Q: Unnecessary prescription of antibiotics.

R: by the Clinicians. Most of us, when I say Clinicians I am involved as well.

Q: Any other factor?

R: yes, aah, incompletion that’s why I said most of the patient do not complete the antibiotics. They just take them for few days and then they leave the drugs so that can also bring resistance.

Q: umhu, any other factor?

R: Yes, I think the major ones are these one.

Q: So you mentioned factors that lead to antimicrobial resistance unnecessary prescription of antibiotics, incompletion of antibiotics by whom; patient?

R: yes by patients. And again by Clinicians because you can prescribe, for example, that condition needs 30 capsules of Amoxicillin and you give a patient 12 definitely the patient will complete but it is you who have given not enough medications to the patient so in such a way even Clinicians can also contribute to the resistance

Q: Okay, Clinician as well.

R: That is under treat or what, yeah I think that is.

**Q: okay, there is any other factor?**

R: (silence) so far those are the only factors I have remembered.

Q: okay, so you have mentioned unnecessary prescription of antibiotics, incompletion of antibiotics by patient, under treatment by Clinician to patient.

R: yeah

**Q: okay. So whose responsibility is to resolve this problem?**

R: Both

Q: whom and whom?

R: The medical personnel’s as well as the patient.

Q: okay, why?

R: We as medical practitioners we need to give the right quantity of drugs to the patient; we need to give the antibiotics to the right condition not any other condition is equal to antibiotics. In so doing we will also prevent the antimicrobial, the resistance and again we have to educate the patient. We have to tell them that they need to complete course given that is if they stop taking medication when they feel they are cured leaving the other tablets or capsule definitely they also enhance the microbial resistance. So that’s why I am saying it’s for both the patient and the clinician.

**Q: umh, okay yeah. Do you have any more addition before we close?**

R; yeah, I think we are as I have said hospital s are also contributing much because the other cases in the hospitals, that are not supposed to be given antibiotics.

Q: umh,

R: yeah

Q: ojkay

R: yes

**Q: Do you have examples of…**

R: Yes, I will tell you of burns, burns. Almost all burns are given antibiotic whether superficial or what you give antibiotic but what does the protocol says you don’t have to give antibiotic to each and every patient who has sustained the burns except when they have a systemic infection. So I think that is contributing to different antibiotic, antimicrobial resistance.

Q; okay

R: yeah, I think that’s what I noted and you know there was a discussion in morning handover about that. I was no we don’t have to give antibiotic to every patient with burns but others said no let’s give. So I think sometimes we don’t know what to do so when you are a lone and there is a group some where you try to talk and argue with them then you just agree that’s okay fine that’s okay. That’s another thing, that’s another challenge.

Q: okay, do you have any additions to add on this interview; pertaining to interview that we are still recording?

R: aah, no

Q: okay

R: yeah

Q: Thank you sir for participating in the study as I have said we will keep this aah, secret. Thank you.

**END OF INTERVIEW**
