# Supplementary material for: Determinants of clinician and patient to prescription of antimicrobials: Case of Mulanje, Southern Malawi
Source: PLOS Glob Public Health. 2022 Nov 16;2(11):e0001274. doi: 10.1371/journal.pgph.0001274 (PMC10022363; doi:10.1371/journal.pgph.0001274)
Supplement: S3 Text — (DOCX) [file pgph.0001274.s004.docx]

**3. Appendix :3, In depth interview with clinician number 3, on determinants of antimicrobial prescriptions in Mulanje District, Malawi.**

I am Maurice Chalusa, I am working with Mulanje district hospital I am also a student at a college of medicine doing Master of Science in antimicrobials stewardship: master of health sciences in antimicrobial stewardship. I am doing a study called ‘Determinants of decision between a Clinician and a patient to prescribe antimicrobials. So I have a questionnaire which will ask you questions. Questions that you feel are not necessary you are free not to answer them. If you feel that the interview is not useful to you, you are free to terminate (clears the throat). The recording of this interview will be kept in a secret place. You are free not to mention your name.

**Q: What is your role at this hospital?**

R: Clinical Officer

**Q: Where do you conduct the majority of your work?**

R: Pediatric ward.

**Q: Pediatric ward. Do you prescribe antimicrobials both antibiotics and antimalarial?**

R: yes, I do.

**Q: okay, which one do you prescribe most; antibiotic and antimalarial?**

R: Antibiotics; Benzylpenicillne, Antimalarial LA.

**Q: So between the two antibiotics and antimalarial which do you prescribe most?**

R: Antibiotics

**Q: Why do you think antibiotics; why do you prescribe most antibiotic?**

R: aah,because ahh, most of the time like ion pediatrics, children are preserved with co-infection like maybe they have come with malaria but you found out they are also showing signs of infections.

**Q: Okay, how many times per day do you prescribe antimicrobials: both antibiotics and antimalarial?**

R: aah, maybe 7 times.

**Q: okay. Share me with what you know about patient factors that influence antimicrobial prescription; what do you think are the patient factors that will influence you to prescribe antimicrobials?**

R: okay, aah, when the patient has presented with symptoms like fevers. Yeah and as I have said that most of the times like in peadiatric we receive a lot of children with malaria but you found out that you have put them on antimalarial but still the condition is still not improving despite that the child is on antimalarial. So you tend to start the child on antibiotics.

Q: okay, any other factor?

R: aah, no.

**Q: okay. so the factors can be in both in adults or in children or in maternity or surgery?**

R: okay, aah like in maternity most of the times we look at the patients we see in terms of hygiene so yo think that this patient maybe is at risk of having bacteria.

**Q: okay, any other factor?**

R: aah, okay like any patient who has presented with a wound we may also think of giving the patient antibiotics.

Q: okay

R: yeah

Q: there is any factor you want to add?

R: ah I think so far I can end there.

Q: okay. so on patient factors you have mentioned of fever , you have also mentioned of a condition when a patient is not improving on antimalarial, you think of giving antibiotic. You have also mentioned of hygiene those people that don’t have hygiene you prescribe antimicrobials those also that presented with wound.

R: eeh

**Q: When did you start prescribing antimicrobials?**

R: it has to be 2018

**Q: what problem do you face during this period when you have started prescribing antimicrobials; what are the problems?**

R: okay, the problems I faced are like maybe you may start the patient on antimicrobials but still you out 2 or 3 days after the patient has been initiated on antimicrobials still the symptoms you were seeing on the patient that made you to start on antibiotics they are still there so sometimes you get stuck like what is going on.

**Q: any other problems?**

R: okay, aah sometimes like more especially Benzylpenicillne in which the dosing is QID most of the times, you found out that the nurses on the ground they may miss other doses.

**Q: okay, any other problem?**

R: aah, I think so far that’s it.

Q: okay so the problem you have mentioned; not improving on antibiotics when the patient you have initiated has a problem. You have also the QID dose not being given to patient from the nurses.

R: yeah

**Q: okay. So what does you patient believe about antimicrobials; what are their beliefs about antimicrobials, your patient?**

R: aah, I should say that most of these patients they don’t know what we are giving them that they are antimicrobials so if they are not aware of the antibiotics, they can’t be aware of the drugs that we are giving most of them.

**Q: any belief?**

R: on the patients?

Q: umh

R: Okay, aah, I think most of the patients they believe in injectable drugs than pills. They believe that you are going to inject them they are going to get healed.

**Q: okay, anymore beliefs?**

R: ah, no.

Q: okay so they believe to say that most of them they don’t know the antibiotics, they also believe in injectable.

R: aah

**Q: okay. suppose you are in outpatient department, then you have tested the patient and see MRDTs negative you did a full blood count, the blood count is normal, what would be the challenge in terms of antimicrobials prescription to you a Clinician?**

R:

**Q: or in General what are the challenges in terms of antimicrobial prescription that you encounter them each and every day?**

R: okay, alright. Most of the times you may found out you have meet a patient as you have said MRDTs is negative and the full blood count shows that the WBCs are within the normal range. But still patient is showing signs that may made you think that this patient has an infection so it comes a challenge. So most of the times we end up prescribing antibiotics.

Q: umh

R: Yeah

**Q: Any other challenge?**

R: okay, aah, like sometimes when you are managing a patient of which maybe is staff or whatever you have met such cases, you end up prescribing antibiotics.

Q: okay

R: so that maybe the patient should feel that you have helped him or her.

Q: okay

R: yeah

**Q: any more challenge?**

R: aah, no.

Q: so I may take you challenges you have mentioned that whenever you meet a staff, you prescribe antibiotics so that you feel you have helped.

R: mmh

Q: and when results are negative you also prescribe antimicrobials.

R: yeah

**Q: okay. In your view, how do you describe the attitude of your patient when you have refused to prescribe antimicrobials?**

R: can you come again.

**Q: in your view or in your experience how do you describe the behaviour or the attitude of your patients when you refuse to prescribe antimicrobials?**

R: I think they feel sad. They feel like you have not helped them.

Q: they feel sad or you are not helping them

R: eeh, sure.

**Q: Any other attitude?**

R: sometimes, they feel like you are incompetent.

Q: umh, they feel like you are incompetent.

R: yeah

Q: any more?

Rr: aah, no

Q: so you mentioned that attitude, they feel sad, that you have not helped them or they also feel that you are incompetent.

R: yeah, sure.

**Q: okay, what communications skills are needed when you are prescribing antimicrobials to patients?**

R: okay, aaah, I think the first you have to explain to the patient the condition she is having or he is having and what drugs you want to give to her and why you are giving that drug and how that drug is going to help that patient. And also we also have to give them the like side effects of the drug which patient is going to face so that the patient should be aware and should be assured.

**Q: any communication skills?**

R: I think thus how we do it here

Q: oky so you have mention that aah the communication skills that are needed are to explain the condition to the patient, you have also tell what type of drugs your patient is getting giving, you have also mention why are you giving that drug, you have also mention how that drug will help, you have also mention how the side effects of the drug, thus what you have explained?

R: yah

**Q: oky how much time do you spend with your patient?**

R: oky, aah in average I should say I spend maybe ten minutes

Q; oky, why do you spend ten minutes?

R: ok, aah coz most of the times I have to even know my patient, yah like what is going on with her yea sure, aah I have to start review the patient might be treated maybe before eeh maybe with another drug so I have to go through the notes and later on come up with my own decision yah.

**Q: so how does this affect your aah, how does this affect antimicrobial prescription to you, the time that you have mention ten minutes how does it affect antimicrobial prescription?**

R: (silence) ah (silence) aah I think it doesn’t affect me in any way

**Q: oky, can you describe guidelines that are used during prescription of antimicrobials aah, both antibiotics and anti-malaria per clinician, the guidelines that you know that you use ?**

R: oky, aah we use the aah Peadiatric white book so far that what we have used, we also have the Malawi standard guidelines MSTG and aah peadiatric pink book by Kazembe

Q: umh

R: yah
Q: any more guidelines that you know?

R: oky, aah (silence) that I know or that I have used?

Q: that you know, that technician use.

R: aah, aah, (silence) have just forgotten the name, we also have the blue book

Q: ok

R: yah, blue book

Q: its, what do they, just blue book or does what, a certain?

R: aah (silence) it’s a clinical book, bleu book

Q: any more guidelines?

R: aah (silence) I think the other have just forgotten

Q; ok

R; yah

Q; so you have mention the guidelines that there is Peadiatric white book, The Malawi standard guideline treatment book, the peadiatric guide book by Kazembe and the Kidney blue book.?

R: yah

**Q: ok, have you ever heard of aah bacterial resistant?**

R: yes I ever heard

**Q: what is it?**

R: aah bacterial resistant in my own way aah in my understanding aah I think this is the state in which bacteria are resisting to the drugs that you are prescribing at that moment.

Q: ok

R; yah

**Q; example, do you have example of bacteria resistant?**

R: those that resist?

Q: umh

R: aah no.

**Q: you don’t have examples?**

R: yah

Q: ok, example of bacteria that are resistant to bacteria?

R: the antibiotic that are resistant?

Q: umh

R: aahoky, aah I have heard of benzlypenecillin and also the Cefriaxone

**Q; ok, so what is meant by antimicrobial resistant?**

R: what, what is what?

**Q: meant by antimicrobial resistant?**

R: oky, it means that aah the microbes, yah they are not susceptible to the drugs that we are giving so they are resisting to respond.

Q: ok

R: yah

**Q: can you describe factors that lead to antimicrobial resistance?**

R: that amy lead oky,

Q: factors that may lead to antimicrobial resistance

R: okay ,aah I think viewing antibiotics when it’s not necessary to give the ant microbes they lead to microbe resistance

**Q; any more factors?**

R: and also aah drug ideas they are some patients they may just take drugs for just a day, and stop maybe they also start after some days the same drug so which may also re do resistance.

Q: any more factors?

R: (silence) I think thus the ones that I know

**Q: so giving antibiotics when its not necessary, aah not finishing drugs it also leads to resistance as you mentioned ,,so what do mean when you say giving antibiotics when not necessary ?**

R : okay I mean like the patient , the client has no indication for ant microbes so you are still giving the patient ant microbes.

**Q: oky who is the responsibility to resolve this problem?**

R: the what?

Q: the antimicrobial resistant problem, who is the responsibility to solve this?

R: I think it us the workers

**Q: why?**

R: coz we are the one who are prescribing this drugs

**Q: oky, how can we resolve this problem?**

R: aah I think aah we have to be aah honest with our penitents and we have to follow what the guidelines are saying on how we should prescribe this drugs

Q: there is anything that you want to add from this interview?

R :aah nothing its oky

Q; thank you, your voice will be kept safe not to be shared with other people , thank you for participating in this study.
