# Supplementary material for: Determinants of clinician and patient to prescription of antimicrobials: Case of Mulanje, Southern Malawi
Source: PLOS Glob Public Health. 2022 Nov 16;2(11):e0001274. doi: 10.1371/journal.pgph.0001274 (PMC10022363; doi:10.1371/journal.pgph.0001274)
Supplement: S4 Text — (DOCX) [file pgph.0001274.s005.docx]

**4. Appendix 4, In-depth interview with clinician number 4, on determinants of antimicrobial prescriptions in Mulanje District, Malawi.**

Q: Good morning my name is Morris Chalusa technical officer of Mulanje District Hospital, am also a student at College of Medicine University of Malawi. I am doing masters of Science in Health Science and microbial (stewardship) so I am doing a study called determinants of decision between a technician and a penitent to prescribe anti-microbial, technician perspective. You are fee not to mention your name in this conversation. Question that you think are edited you are free not to answer them, you are so free to terminate this recording any soon you feel are not comfortable to proceed with the interview.

Q: so can we proceed?

R: Yes

Q: What is your work in this district hospital?

R: I am a Healthcare Practitioner at (not clear) medical assistant

Q: Ah where do you conduct majority of your work?

R: I conduct majority of my work at out penitent department

Q: Do you prescribe antimicrobials?

R: Yah

Q: which one do you prescribe most?

R: Mostly its Amoxicillin, Cotrimoxazole, Metronidazole, Doxycycline mostly are those.

Q: How many times a day do you prescribe antimicrobials?

R: Ah, almost any time.

Q: any time?

R: yah

Q: You said you do prescribe antimicrobials?

R: Yah

Q: aah you mention about Amoxilline and Cotrimoxazole?

R: yah

Q: so in terms of other type of antimicrobials like anti-malaria, how many times a day do you prescribe?

R: ah any time.

Q: Why do you think you prescribe these anti-microbial anytime?

R:ah so far patients usually come and when I diagnose the patients regarding to their clinical presentation, yah according to diagnosis

Q: Can you share me with what you know about patient factors that influence anti-microbial prescription ,when you are at the OPD what do you think are some of the patient factors that will influence you to prescribe anti-microbial ?

R: mmm, mostly patient expectances; whenever they are coming with a slight complaint they do think they are going to get anti-biotic.

Q: ok, any other factor that will influence you to prescribe anti-microbial to patient?

R: Concerning to the clinical scenario to the patient, other patients may look so ill with fevers and chill but when you test them maybe for malaria, you find that they are maybe negative so may attempt that it might be sepsis and then prescribe the antimicrobials

Q: any other factor that influence you to prescribe anti-microbial to a patient?

R: they are those

Q: okay so you have mention factors that influence to prescribe antimicrobial: Patient expectation; when patients are coming to the hospital they do expect that you will prescribe anti-microbial, thus what you are saying. You also mention that clinical presentation makes you to prescribe the anti- microbial and you also mention that when MRDTs is negative it also influence you to prescribe anti-microbial simply because the patient might have sepsis.

R: yah, but have said the first one on expectation not really.

Q: ok, when did you start prescribing the antimicrobials?

R: soon after starting our internship.

Q: ok, soon after internship particularly when?

R: September 2018

Q: So since September 2018 what problems do you face during this period when you are prescribing antimicrobials?

R: mostly when patients come they turn to worry and complain when they are not given the drugs they were hoping for like antimicrobials, some may have flue and they expect that you will give them an antibiotic.

Q: ok, any other problem?

R: No

Q: so you have stated that the most problem that you are meeting is patient worry, when they are not given anti-microbial they want.

R: and also you may find out that other patients are used to get antibiotics but there is no improvement

Q: ok, you have any other problem you are facing when prescribing anti-microbial?

R: No, they are those

Q: Can you explain to me your thought regarding to patients believe regarding to antimicrobials, what does your patients believes about anti-microbial.

R: many patients believe that whenever they are given antimicrobials they are going to be cured from what they are suffering from.

Q: any other belief?

R: mostly they turn to go for injectable, whenever they come to the hospital they complain that they should also get the injectable.

Q: any more belief from your patients?

R: no

Q: So you are saying that your patients always believe that when they are coming to the hospital and are given anti-microbial they get cured?

R: yah

Q: They also believe that they are going to get injectable?

R: Yah

Q: you also mentioned the other point?

R: No

Q: They are two points?

R: yah

Q: What challenges do you encounter when you are prescribing antimicrobials such as (antibiotic antimalarial) , suppose you are at the OPD so you test them and it is negative MRDTs, what are challenges that you are facing in kind of situations?

R: mostly you find out that when you give patient maybe Analgesics, they complain that they need antibiotics, and also maybe thinking of ……yah but so far they complain mostly.

Q: they do complain about what?

R: like I stated earlier that they are not helped as they need antibiotics.

Qr: okay, any other challenge?

R: no

Q: Okay, so you say challenges are; most of them want antibiotics and other complain that you have not help them when you have given them analgesic instead of antibiotics, okay do you have any point to add on this?

R: no

Q: in your view how you would describe the attitude of your patients when you refuse prescribe antimicrobials.

R: mostly they react as if I did not went to medical school, like I have just been taken from somewhere, they do just think that am not a medical practitioner, that am not well equipped in knowledge and sometimes they turn to be saying that this one is just small concerning knowledge, they do go for, some do talk that am just small and the knowledge is just narrow.

Q: Any attitude from your patients? Thus all?

R: yah

Q: so you say that the attitude is that they say you are not medical practitioner, they do also say that you are not equipped in knowledge, the other point you say what?

R; it’s concerning my age

Q: you are too young to them?

R: yah

Q: what are communication skills needed for prescribing antimicrobials? what communication skills are supposed to be there when you arte prescribing anti-microbial?

R: to have an awareness campaign to the patients, to tell them that an antibiotic is not usually meant for any illness.

Q: any communication skills?

R: Concerning the duration of taking the antibiotics, they should take as prescribed by the medical practitioner.

Q: any more communication skill?

R: no

Q: so you said the communication skills that are needed are awareness that an antibiotic is not for any illness, you also mention the duration of taking an antibiotic.

R: Yah

Q: okay can we proceed?

R: yah

Q: how much time do you spend with your patients?

R: mostly its aah, concerning maybe with the condition, we spend about five to eight minutes in the OPD.

Q: so you are saying it all depends?

R: yah

Q: how does this affect the anti-microbial description?

R: when you take a long time with the patient many turn to understand the treatment you are giving them

Q: can you describe some of the guidelines that are used during the prescription of antimicrobials, antibiotic and ant malarial by technician?

R: like the books

Q: yah, the guidelines can be the books, posters being put in the walls

R: Paedriactics book, the BNF and also MSTG and there is also TB guideline 2018 and also HIV related cases guideline 2018, the BNF, Malawi standard treatment guidelines, the TB and HIV guidelines?

R: yah

Q: any more guidelines?

R: and also those posters at OPD.

Q: okay can we proceed?

R: yah

Q: Have you ever heard of anti-microbial resistance?

R: yah sure

Q: what is it?

R: It is the situation where by antimicrobials, the microbes now they do overpower the drugs, yah, whenever the patient take drugs they don’t work.

Q: okay, so you are saying antimicrobial is the resistance from the microbes, whenever the patients take drugs they don’t work.

R: yah

Q: could you have the type of bacteria that are resistance to antibiotics or do you have practical examples of antibiotics that are resistant to bacteria or have you ever heard of any antibiotics which is not working against bacteria?

R: mmm yah, like Benzylpenicillne, ampicillin, Amoxicillin and even Ceftriaxone

Q: In one word what it means anti-microbial resistant?

R: It is when the microbes do not respond to the antibiotics

Q: thus all?

R: Yah, it is when the microbe do not respond to anti-microbial therapy, yea thus all.

Q: Can you describe some of the factors that lead to anti-microbial resistance? Antibiotic and anti-malaria

R: under prescription,

Q: under prescription, mh

R: lack of sanitation

Q: lac k of sanitation mh

R: and also over prescription

Q: over prescription of antibiotics?

R: yah

Q: any?

R: ah no

Q: so you mention that factors that lead to anti-microbial resistance is under prescription, lack of sanitation and over prescription on antibiotics?

R: yah

Q: so whose responsibility to resolve this problem?

R: I think the physician and also the patient

Q: ok

R: both

Q: ok, physicians and patient?

R: yes

Q: why do you think physicians are responsible?

R: physician he should be able on prescribe the antibiotics, to prescribe the whole dosage and also to advise the patient on the important of taking the whole dosage in a right duration, yah

Q: ok, why the patient has the responsibility to resolve this problem?

R: for their maximum recovery, with the right duration will eradicate the particular pathogens, so for their good recovery they should be able to take the whole prescribed dosage.

Q: thank you sir thus the end of our Questions, do you have anything to add?

R: No

Q: ok, thank you the recordings will be safe they will be kept in a computer in a secrete way no one will have access to it.
