# Supplementary material for: Determinants of clinician and patient to prescription of antimicrobials: Case of Mulanje, Southern Malawi
Source: PLOS Glob Public Health. 2022 Nov 16;2(11):e0001274. doi: 10.1371/journal.pgph.0001274 (PMC10022363; doi:10.1371/journal.pgph.0001274)
Supplement: S5 Text — (DOCX) [file pgph.0001274.s006.docx]

**5. Appendix:5, In-depth interview with clinician number 5, on determinants of antimicrobial prescriptions in Mulanje District, Malawi.**

AQ: Good afternoon sir?

R: good afternoon sir

Q: My name is Morris Chalusa, am a clinical officer working at Mulanje hospital I’m also a student at University of Malawi College of Medicine doing Masters of Science in Health sciences .As a requirement for the degree I’m supposed to do a study, so choose to do this study in Mulanje district hospital and Mulanje mission hospital and health centers. My study is determinant of decision between technician and patients to prescribe antimicrobials clinician perspective. You are free to terminate this interview this recording whenever you feel you are offended or if you think the questions are not appropriate, you are also free not to answer some other questions that irritate or not important to you. You are also free not to mention your name in this recording. The interview will be kept safe, no one will be able to access it apart from me and my supervisor.

R: ok

Q: Can we proceed?

R: yes we can proceed

**Q: What is your role at this District Hospital?**

R: I am a medical assistant here.

Q: thank you

R: yes : Where do you conduct majority of your work?

R: at the OPD department.

**Q: do you prescribe antimicrobials, both antibiotics and anti-malaria?**

R: yes I do.

Q: ok, which one do you prescribe most, antibiotic and anti-malaria?

R: Amoxicillin and LA

Q: why do you think you do prescribe Amoxilline and LA?

R: because they are a lot of cases of malarias in our City but also a lot of conditions that affect the respiratory systems and so Thoth.

**Q: How many times a day do you prescribe antimicrobials?**

R: fifteen times a day.

**Q: Which one are the most prescribed between anti-malaria and antibiotic?**

R: the most are the antibiotics

Q: ok, can we proceed?

R: Yes we can

Q: OK

R: yah

**Q: share me what you know about patient factors that influence anti-microbial prescription when you are at the OPD, what you think are the patient factor that will influence you to prescribe the antimicrobials.**

R: okay, it’s about the patient preferences

Q: okay

R: yah

**Q; what you mean when you say patient preferences?**

R: okay, it’s just like there are a lot of antibiotics but patients may choose that I like this antibiotics, when I take it I feel good I recover from my complaints, my disease.

Q: ok

R: yes

**Q: any other factor that influence you to prescribe the anti-microbial to patient?**

R: yes, could be just patient expectation according to clinical condition of her disease

Q: ok, patient expectation?

R: yah

Q: ok, so patient expectation and condition of her disease the same or different?

R: it’s different

Q: so you mention patient preferences, expectation of patients, and condition of disease. ?

R: yes

**Q: Any other factor that influence you to prescribe anti-microbial to patient?**

R: no

Q: when did you start to prescribe the anti-microbial?

R: we prescribe if we diagnose the patient’s problem is coming due to bacterial infection.

Q: okay, when did you start prescribing anti-microbial?

R: in 2017

Q: ok

R: yah

**Q: so during this period, what problems do you face during this period when you started prescribing anti-microbial?**

R: problems can you explain?

**Q: what problems do you face when you are prescribing anti-microbial since you started?**

R: ok, maybe others they are not recovering from their own disease they are also coming again to the hospital.

Q: not recovering from their disease, any other problem?

R: other problem is that, shortage of the antibiotics

Q: any other problem?

R: yes, it’s like the patients they misuse the antibiotics

Q; ok

R; yes

Q: shortage of antibiotics, any other problem?

R: No

Q: so you have mentioned that the problem you are face when you started prescribing the anti-microbial is that patient are not recovering , as a problem, shortage of anti-microbial, you also mention misuse of antibiotics?

R: yes

**Q; ok, so suppose you are at the OPD, you are working and you have a patient and you have tested patient and patient result have detected negative MRDTs. You also do full blood count and you find out that there full blood count is normal. Can you explain to me your patient’s belief about anti-microbial?**

R: okay, they believe that they can get better on their condition if they can be prescribed for antibiotics.

Q: ok, any other belief?

R: they also just think that when they go to the hospital ant given the antibiotics they think that they have been given strong treatment, best treatment.

Q: ok, any other belief?

R: the patient may also believe that it takes a long time for them to suffer again if they have been prescribed with antibiotics.

Q: any other belief?

R: no

Q: so you say patients believe that they will get better when they are given antibiotics?

R: Yes

Q: they also believe that when they are given antibiotic they have been given strong antibiotic.

R: Yes

Q: they also believe that when they are given antibiotic it will take time to have the problem again.

R: yes

**Q: in your view how do you describe the attitude of your patient when you refuse to prescribe the antibiotic? Suppose the patient has come, you have tested everything and its normal and you refuse to give drugs.**

R: yes

Q: yah, what’s the attitude to you, how do they feel about you?

R: they feel as if you are stingy for the drugs

Q; okay

R: yah

Q: what you mean when you are saying stingy?

R: okay, you don’t want to give then the better medication, the strong medication or they just think that you have misdiagnose them thus why you didn’t give them antibiotics

Q: any other attitude?

R: they also think that you don’t know your professional you don’t know how to do your work. Let’s say you have given someone a PCM they say that you have not help them

Q: any other attitude of your patients when you refuse to prescribe antimicrobial, both antibiotics and anti-malaria?

R: no

Q: so you have mention that the attitude are; they feel that you misdiagnose them, they feel that you did not give them strong medication they also feel that you don’t know your work.

R: yah

**Q: ok, what communication skills are needed when you are prescribing antimicrobials to a patient, what communication skills are needed when you are prescribing the anti-microbial?**

R: it’s to ask the patients if they know or what they know about antibiotic or how they can use the antibiotic.

**Q: any other communication skill?**

R: of course yah, we have also to tell the patients how they can use the antibiotics, according to the parodist

Q: ok?

R: yes

Q: any other?

R: But also just to tell them the side infects of the drugs

Q: ok?

R: yes

Q: any other?

R: no

**Q: how much time do you spend with each patient when you are prescribing the antimicrobials?**

R: about two minutes

Q: why do you spend two minute to this patient you have prescribe antimicrobials?

R: ok, because they are a lot of patients at the Out Patient Department

**Q: so how does this affect your work as in prescribing of antimicrobials?**

R: this affect me as I don’t have much time to do other further investigation per each patient

Q; OK

R; yes

**Q: can you describe some of the guidelines that are used during prescription of antimicrobials, antibiotics and antimalarial by technician?**

R: can you come again?

**Q: can you describe some of the guidelines that are used by the technician during prescription of antimicrobials?**

R: yah, some of the guidelines like in STIs we use triple therapy like to give drugs like gentamicin, metronidazole but also DCN

Q: okay

R: yah use this guidelines but also they are some guidelines for other patient that have…for HIV patients that have opportunistic diseases like the Cryptococci meningitis they are guidelines that we use. We use newly guidelines one like the use of amphotericin B but also cytosine

Q: okay

R: the first yah

Q: any other guidelines that are used?

R: no

**Q: Have you ever heard of antibiotic resistant?**

R: yes

Q: what does it mean by antibiotic resistant?

R: ok, antibiotic resistance it’s when the bacteria is not able to be defeated by the antibacterial prescribed

**Q: do you have example of antibiotics that are resistant to bacteria?**

R: yes, like Cotrimoxazole, amoxicillin.

Q: ok, do you have any other antibiotics?

R: we have the fluroquinones one like ciprofloxacin they also build resistance to other bacteria.

Q; ok, any other antibiotic?

R: we have metronidazole the {anural} one

Q: ok, any other antibiotic?

R: yah, they are a lot of them like penicillin ones like flucoxacilin

Q: ok

R: yes they also build resistance

**Q: what is meant by antimicrobial resistance?**

R: what?

Q what is meant by antimicrobial resistance?

R: meat?

**Q: what is the meaning of antimicrobial resistance?**

R: ok, antimicrobial resistance which means that the bacteria is not responding to the antibiotics we are prescribing.

Q: ok

R: yah or we can say that if you are giving the patient drugs with certain bacterial infections are not responding to that particular drug.

Q: ok

R: yah, means that the bacteria has fail to respond to the drugs we are prescribed or is just like it has build resistance not to be defeated by the antibiotics we are giving.

**Q: can you describe some of the factors that lead to antimicrobial resistance?**

R: yah, some of this factors could be under dose, if you don’t give adequate dosage to the patient can build the resistance because the under dose cannot be able to fight against bacteria.

Q; any other factor?

R: yah but also the, can say if the the patient has not finish the drugs can also build the resistance. But also the other factor can be using the antibiotics not necessarily to be used as maybe patient condition is not about bacterial infection and if you are giving antibiotics it can also build resistance.

Q: ok

R: yes

Q: any other factor?

R; no

Q: ok

R: yes

**Q: who is responsible to solve this problem, antibiotic resistance?**

R: we as medical professionals we are responsible to solve this problem.

Q: ok

R; yes

Q: why?

R: because we are the one who are prescribing the antibiotics to patients

**Q: There is anything that we have to add? , we have reached to the end of our interview.**

R: Yes, according to the issue of antimicrobial resistance, maybe we have to alert one another as medical professionals to the proper diagnosis for antibiotics to be prescribed because some time we may just give the antibiotics not necessarily that the patient has got a bacterial infection , we as professionals we have to alert one another or teach one another. But also we need to have a health talk through the primary to tertiary education that when the clinician or the physician have prescribe the drugs they have to accept because are the one that have knowledge.

Q: thank you sir, thus the end of our interview your data will be in a safe will not be revealed to anyone else

R: thank you.
