# Supplementary material for: Determinants of clinician and patient to prescription of antimicrobials: Case of Mulanje, Southern Malawi
Source: PLOS Glob Public Health. 2022 Nov 16;2(11):e0001274. doi: 10.1371/journal.pgph.0001274 (PMC10022363; doi:10.1371/journal.pgph.0001274)
Supplement: S6 Text — (DOCX) [file pgph.0001274.s007.docx]

**6.APPENDIX:6, In-depth Interview with Clinician Number 6 on determinants of antimicrobial prescription in Mulanje District, Malawi.**

Q: Good afternoon, sir

R: good afternoon

Q: I am Morris Chalusa a clinical officer working with Mulanje hospital in also a student at College of Medicine University of Malawi doing Master of Science antimicrobial stewardship, ah I’m doing a study called determinants of decision between clinician and patient to prescribe antimicrobials clinician perspective. Aah you are free to terminate this recording any time you feel that you have been offended, you are fee not to answer a question that you think are irritable that are not appropriate to you. Be free you are also free not to mention your name in this interview. Thank you.

R: welcome

Q: can we proceed?

R: yes

**Q: what’s your role at this district hospital?**

R: aaa I am a clinical officer

Q: ok, where do you conduct majority of your work?

R: Maternity

**Q: do you prescribe antimicrobials both antibiotic and anti-malaria?**

R: Yes I do

Q: (clearing throat) aah which one do you prescribe most? Between anti-malaria and antibiotic

R: when comes to anti-malaria its mostly LA but most antibiotics we prescribe is Metronidazole and Ceftriaxone IV and Benzylpenicillne IV in POs we have Metronidazole and Amoxicillin

Q: ok

R: these are the commonest ones

Q: in terms of anti-malaria?

R: anti-malaria it’s LA and SP

**Q: ok, why do you mention those antimicrobials do you prescribe them a lot?**

R: Because they are commonly used in our setting

Q: okay

R: yes

**Q: why the anti-malaria that you have mention?**

R: Because mostly they are the ones like aa guidelines recommends the nation malaria guidelines and also in pregnant women are advisable to give SP

**Q: okay, aah in average a day how many time do you prescribe antimicrobials, both antibiotic and anti-malaria?**

R: in average as a number?

Q: umh

R: it’s almost Fithteen –twenty

**Q: ok, which one do you think you prescribe a lot between antibiotic and anti-malaria?**

R; the antibiotics are the ones

Q: why antibiotics?

R: mostly because we deal with aah bacterial infections than the parasites, so we prescribe mostly the antibiotics.

**Q: ok, share me with what you know about patient factors that influence antimicrobial prescription when you are in your health care settings.**

R: umh

Q: okay, what factor you think they will influence a technician to prescribe antimicrobial, factors come from patient?

R: aah sometimes aah patient do have their preferences on which drugs antibiotics to be prescribed to them which express or sometimes to them that they feel that its best for them not the condition they have, what’s best for them.

Q: mmh

R: yees, aaand sometimes the influence mostly the patients do have its makes us to prescribe aah certain antibiotics.

Q: mmh (clearing the nose pipe)

R: and sometimes we look also at the (silence) pharmacological factors like how the drugs works between the pharmacodynamics and the pharmacokinetic effect, it’s what mostly does a lot ,soooh yah mostly those and also maybe the duration of the aah period to be taken while taking the antibiotics for the patient, so they don’t to be burdened most of the time so they choose antibiotics which can only take them short period of time.

Q: oky?

R: yah, sooh which to them seems not to have aah much side effects.

Q: mmh

R: yes

**Q: Any Patient factor that will influence you to prescribe antimicrobials?**

R: So, factors will depend with thu condition of the patient

Q: mmh

R: yes, (silence) cause if it’s a severe infection then I think tells us to which drug to give mostly we go for strong antibiotic and at time we use those that are available in our pharmacy and also at times ah (silence) we just use to prescribe ah a specific antibiotic rather than even though we have so many option at the table.

**Q: ok, do you have any factor, any more factors?**

R: No

Q: ok, so you described quit a wide of factors ah that influence antimicrobial prescription, from the patient you say ah some patients have antibiotic preferences, they want specific antibiotic to be prescribed. Sometimes you prescribe antimicrobial because patient has influenced you; you also said pharmacological factors also influence you to prescribe antimicrobial to a patient. You also mention period of taking antibiotics including side infects, you also mention the condition of a patient can influence you to prescribe antimicrobials and you also mention availability of antimicrobials at a pharmacy.

R: yes

Q: can I proceed?

R: yes

**Q: when did you start prescribing antimicrobials?**

R: I started in 2017

**Q: what problem do you face during this period when you started prescribing antimicrobials?**

R: ah the problems that are faced mostly is that ah we are limited when it comes to which drugs to give to the patient based on what ah is available.

Q: mmh

R: sooh, Mostly you find that we repeat the same antibiotic to a patient for a longer period of time because we do not have any other alternative, so it’s like a problem or challenge to us when it comes to prescription of the antibiotics.

**Q: ok, any more challenge?**

R: aaah, no

**Q: okay, (silence) can you explain to me what thought regarding to patient believes about antimicrobial, what do you think patient believes about antimicrobials, what do they believe about antimicrobials?**

R: yah

**Q: when the patient has come to the hospital, what do they believe about antimicrobials?**

R: so, most patients believe in that whenever they come to the hospital, they have to get the drugs and to go home with the drug antibiotic even if the condition doesn’t fit for them to take the antibiotics and as I have said earlier mostly it’s their preferences which they do like or recommend themselves so when you try to prescribe to them another type they show some resistance so thus one of the (silence) things.

Q: okay

R: yah

**Q: any more beliefs? (silence) what would totally do believe when they have come to the hospital and you have examined them and you are giving instructions about antimicrobials what do they believe?**

R: sooh, mostly if they believe in to say that ones they feel better they will stop the antibiotic even if the duration of the ah, ah, dosage isn’t yet finish they will still stop the taking antibiotics simply because they are feeling better for that moment.

Q: oky

R: yes

Q: can we proceed?

R: Yes

Q: so in terms of belief you are mention that aah, (silence) patient do come to the hospital to get antibiotics although the condition they have a condition that do not fit to have the antibiotics, you also mention that patients have their preferences in terms of antimicrobials, you also mention that patient will stop taking the antibiotics when they feel that they are better without finishing their dosage.

R: yes

Q: okay, suppose we are in outpatient department in Out Patient Department, so there is bunch of patient come in, you do MRDT negative despite presenting with symptoms of malaria, but you do full blood count its normal, okay?

R: mmh

**Q: what problems do you face to this such kind of patients?**

R: so in those cases the challenge is that most of the patients do come a diagnosis in their head with the way I m feeling it might be imperia so if you do all the examination and other tests and you find out that it’s not malaria they will still insist that they should get ant malaria or even if it’s that they are not fit for antimicrobial, a certain antimicrobial they will still push you to prescribe for them such a medicine so it’s one of the challenge cause if you don’t do that they will label you as a bad doctor .

Q: Okay, any more challenge?

R: aah, no.

Q: so in terms of challenges you say that, aah they will come their mind with already a diagnosis in which they want a particular medication.

R: yes

Q: you also mention about the anti-malaria been given when the results are negative.

R: negative, yes

**Q: so the other challenge they will push you to prescribe the ant malaria and antimicrobial if you refuse it they will label you as a difficult.**

R: yes

Q: so since you have started working what are the challenges that you meet when you are prescribing the antimicrobials?

R: sooh , one of the challenges mostly (silence) its aah repeative usage of single antimicrobial, even in the same patients or in most out patients seen or even in patients, so it’s mostly certain antibiotic dominate over other antibiotics, so that’s one of the challenge simply because it’s it has develop some resistance simply because of overuse and it has cause most of the unfavorable side infects and which are mostly difficult to treat so are some of the challenges we have meet so far.

Q: okay

R: yes

Q: do you have any challenge?

R: aah so far no.

Q: oky, so in terms of challenge you say repetitive use of antimicrobial, which also lead to resistance of use of antimicrobials you have also mention about unfavorable side effects of antimicrobial .

R: yes

**Q: in our interview how do you describe the altitude of your patient when you refuse to prescribe the antimicrobials?**

R: eh, the attitude so bad, mostly have aah a negative attitude on you and they think like you are denying them the medications and you want to save the drugs instead of giving to them and you are taking them to drug store in market to sell but yet they don’t understand so they will label you as someone who is so difficult when it comes to prescription and they will have a negative attitude and you know that next time they won’t be seen by you again.

Q: so you mention that they will have a negative attitude on you, aah they think that you are denying the medications, they like you want to sell the meds to the market, so next time you say they will not come back to you to see them they will go to another one.

R: yes

Q: can we proceed?

R: yes

**Q: what communication tools are needed when you are prescribing antimicrobial?, what communication skills are needed when you are prescribing antimicrobials?**

R: so, mostly its only what’s needed is to create aaah, a rapport between you and the patient so that whatever you are say the patient should have a total trust in you, even if you are say that you not conform with the medicine the patient should be able to understand and also even before prescribing the antibiotics you need to clearly explain to the patient how the drugs works and what are the side effects what are the outcome after all so I think those are the things that, skills that need to, we need to have when, before prescribe the antimicrobials or before seeing our patients.

Q: oky

R: Yah

**Q: do you have any more communication?**

R: no

**Q: so in terms of communication skills you mention of developing rapport with the patient, explain how the drugs work to the patient, explaining the side effects and also explaining to the patient the outcome.**

R: yes

Q: the outcome of the?

R: like after taking the drug what’s expected out of it.

**Q: ok, how much time do you spend with each patient?**

R: aah mostly with the queue that we do have almost five minutes

Q; oky

R: yes

**Q: how does it affect antimicrobial prescription, to you, the time that you have mention?**

R: yah, it affects a lot because you will not have much time to explain to the patient and you will just prescribe antibiotics which as they come to your mind maybe even without thinking of the side effects of the antibiotic or even the mode function of the antibiotic in relation to the what the patient presented on what you have diagnose the patient with.

Q: oky, can we proceed?

R: yes

**Q: can you describe some of the guidelines that are used by the clinician when you are prescribing the antimicrobials?**

R: so, guidelines it’s mostly we use the standard aah Malawi standard guideline treatment, and also we have some guidelines which are now available due to several researches which have been done they have recommended so many things and also we do use the aah BNF, the British National Formula which helps us when it come to the dosing and prescription in relation to the condition of the patient.

**Q: ok, any more guides?**

R: yah we have the (silence) aah hand book which we use when it comes to the prescription and also the aah clinical hand book it’s also used as a guidelines when prescribing the antimicrobials.

Q: oky, should we proceed?

R: yes

**Q: have you ever heard of anti-bacterial resistance, have you ever heard of bacterial resistance?**

R: yes have heard of it.

Q: what is it?

R: so mostly it’s when the drugs have now no effect on the patient or on the aah condition of the disease which the patient presents its when given there is no change simply because the bacterial have develop their own defense to the drug therefore will not work or not give the expected results .

**Q: thank you, so which type of antibiotics have developed into resistance?**

R: so mostly (clearing throat and nose pipe) the ceftriaxone its coz mostly commonly used and aah the metronidazole also developed resistance because of the repeated use.

**Q; Okay, so can you define what is meant by antimicrobial resistant?**

R: (yawning) oky sooo, (silence) this is when aah the antibiotics aah aah failing to…. Work on the. Bacteria that cause infection there by leading to less effect after taking the drugs or after prescription or after completion of the dosage.

Q: oky. Can we proceed?

R: yes

**Q: describe factor that lead to antimicrobial resistance, both antibiotics and anti-malaria?**

R: so the factors mostly its ah the duration of the ah dosage because most of the antibiotics demand that they should be direct almost five to seven days but mostly you find out that the patient is only given aah two days then you its stops ah the drugs and you switch to another drug so that is one of the factors that has aaah is causing resistance and also the other thing is ah the repeated use of drugs ( silence) and the other thing is aah mostly mutation due to the aah patients aah poor compliance to the drugs to the prescribed antibiotics.

**Q: Okay, do you have any?**

R: no
Q: ok, so you have mention of duration (clearing the throat) of the medication being taken by the patient being one of the reason for the resistance, you have also mention repetitive use of drugs, of the same antibiotics can also cause resistance and you also mention of mutation as one of the causes of antimicrobial resistance.

R: Yah

Q: can we proceed?

R: yes

**Q: so whose responsibility to resolve the problem?**

R: sooh, It’s the responsibility of both the patient and the care givers to deal with this problem, once we create an understanding that everybody understand his or her role to play in aah dealing with the resistance thus when I think the solution will be found but it’s both side are involved in the resolution of the problem.

**Q; so aah why do you think both technician and the patient it their responsibility?**

R: yes aah the clinician has the responsibility to the prescription of the drug and the patient has the responsibility to compliance to the given prescription like should follow the aah rules like if it’s for five days the it has to be taken for five days even if the patient starts feeling well even after taking a single dose but has to finish the whole dose so the clinician have to understand how the drugs works in the patient and also explain the condition to the patient and the patient should be able to understand and to be able to compliant to the dosage

**Q: okay, thank you sir for participating in this study, thus marks the end of our interview do you have any additions to add?**

R: aah no thank you
