# Supplementary material for: Determinants of clinician and patient to prescription of antimicrobials: Case of Mulanje, Southern Malawi
Source: PLOS Glob Public Health. 2022 Nov 16;2(11):e0001274. doi: 10.1371/journal.pgph.0001274 (PMC10022363; doi:10.1371/journal.pgph.0001274)
Supplement: S7 Text — (DOCX) [file pgph.0001274.s008.docx]

**7.APPENDIX :7, In-depth interview with clinician number 7 on determinants of antimicrobial prescription in Mulanje District Malawi.**

‘Good afternoon Sir?’

‘Afternoon’

‘I’m Morris Chalusa, a student from the College of medicine, University of Malawi, I’m doing Master of Science in Health Sciences and antimicrobial stewardship. I’m doing a study called DETERMINANTS OF DECISIONS BETWEEN CLINICIANS AND PATIENTS TO PRESCRIBE ANTIMICROBIAL: A CLINICIAN PERSPECTIVE. It’s a quantitative study actually. I have a guide for your interview so in this interview, you are free to participate, you are also free to withdraw anytime that you feel you have been offended, you are also free to not answer any questions that you feel are not relevant to you or they are irritable and you are also free to not mention your name in this interview. The recording will be kept safe and the only person who will be able to access this recording is myself, the principal investigator, my supervisor, and the one who is going to help me in analysing. Thank you.’

‘Thank you, you can proceed’

‘What is your role at this district hospital?’

‘I’m a Clinical Officer.’

‘Thank you. Where do you conduct a majority of your work?’

‘Usually, Male ward.’

**‘Do you prescribe antimicrobials both antibiotics and antimalarial?’**

‘Yes, both. I usually prescribe antibiotics and antimalarial.’

**‘Which one do you prescribe most? The antibiotics and antimalarial?’**

‘Most of the times I prescribe the antibiotics more than the antimalarial’

**‘Why do you think you do prescribe more antibiotics then antimalarial?’**

‘Because I meet more patients who require antibiotics than those who require antimalarial’

**‘On average, per day, how many times do you prescribe these antimicrobials?’**

‘It is usually more than four times a day for the reason that I work in the male ward and we usually make ward rounds so we do have for example in a day, more than 30 patients so we have to do rounds and see each patient and for that reason, we do prescribe more than 4 times a day because we see a lot of cases in a day which require antibiotics.’

**‘Will you share with me the patient factors that influence antimicrobial precribition?’**

‘There are several factors one of which is history which is the presentation of the patient’s condition which must present with symptoms of systemic infection like fever, rates of reduced blood pressure which can be in case of sepsis, raised pulse rate as well as respiratory rate.’

**‘Okay, do you have any more factors from the patient that can influence you to prescribe antimicrobials?’**

‘From the patient, we have other factors like we usually meet other conditions like meningitis where our patients will present signs of meningitis and this usually happens in bacterial meningitis where we have to prescribe antibiotics and patients usually present with severe headache, they also present with neck stiffness, neck pains and they will have a Kenings Sign positive on examination, other cases like neurosyphyllis we also meet those cases several times where we need to prescribe them with Benzathine penicillin for 10-21 days. Those are some of the cases with their signs and symptoms.’

**‘Do you have any factors that can influence you providing antimicrobials from the patient? Anymore?’**

‘Anymore? Of course we have other factors more especially when we do lab tests where they have raised white blood cells which is also one of the signs that the patient is having a systemic infection. Other lab tests we do Lumber Puncture and it’s significant as it is showing glam positive or glam negative causative organisms or diplococcic or any causative organisms. That’s one of the clinical factor that may make me to prescribe antibiotics.’

**‘Thank you. Can I proceed?’**

‘Yes.’

‘so you mentioned history of the patient, presenting condition, fever, reduced blood pressure, increased pulse rate, increased, increased respiratory rate, where you suspected sepsis, meningitis, also presentation on the lab test. All these factors will influence you to prescribe antimicrobials?’

‘Yes.’

**‘Thank you. When did you start prescribing the antimicrobials?’**

‘I started prescribing antimicrobials way back in 2013 when I started working as a clinical officer at Mulanje district hospital.’

**‘What problems do you face during this period when you started prescribing antimicrobials?’**

‘The problems that I have come across or encountered ever since I started prescribing antimicrobials are lack of enough resources to investigate or confirms the diagnosis. For example, the patient will show signs and symptoms consistent with systemic infection but you take specimen, they do not have testing kits at the lab, they are not doing full blood counts, they don’t have reagents to do CSF analysis. Those are some of the challenges we do face.’

**‘Anymore challenges?’ (Problems)**

‘Of course there are other challenges more especially to the patient’s side when the patient will not open up enough. That becomes another challenge as well. Actually, when the patient looks very sick but the patient is not opening up enough to tell you enough history which can help you to come up with a definitive diagnosis. Another challenge can be on the resource part as well, lack of resources especially at the ward. For example, we do always have problems with thermometers, BP machines so we usually have challenges in checking temperatures and blood pressures so that’s another challenge as well.’

**‘Do you have more challenges?’**

‘That’s enough.’

‘So in problems you mentioned lack of investigations whereby there is no … in the lab. That’s what you mean, yes?

‘Yes’

‘Lack of … whereby you want to confirm for a diagnosis, you also mentioned lack of information from the patient, lack of resources like thermometers, BPs and you also mentioned lack of diagnostic equipment in the lab. So in your explanation, you mentioned something like CSF, what is it?’

‘CSF stands for Cerebral Spinal Fluoride.’

**‘Explain to me your thought about patient’s belief on antimicrobial. What do your patients believe about antimicrobials?’**

‘According to my understanding and the experience that I have had, patients’ understanding on antimicrobials is that they believe that antimicrobials especially injectable heal any form of severe illness. Even if it is not a bacterial infection, they still think that if you give them IV and antimicrobials, they are going to recover.’

‘Any belief from the patient’s side?

‘From the patient’s side, that’s enough.’

‘So you are saying that the patients believe that any injectable will heals them? Whether it is an antibiotic or antimalarial? To any form of infection whether it is a virus or a bacteria’

‘Yes.’

‘What challenges do you encounter when you are prescribing antimicrobial whether antibiotics or antimalarial in your everyday life?’

‘We do meet several challenges of course. For example, when we come across patients who are showing signs and symptoms consistent with malaria and when we do malaria check it is negative, they will still insist that they get antimalarial. The same applies to the antimicrobial. If they think that their condition is going to respond to the antimicrobial, they will still want to convince you or to force you, more especially when they are members of staff at the hospital, to prescribe antimicrobial because personally they think that their illness is going to respond to the antimicrobial so it becomes a challenge.’

‘Any more challenges?’

‘Personally, or?’

‘Any challenges

‘Even it can be experiences with other clinicians?

‘Yes.’

‘Of course, other challenges include when a patient comes and you are suspecting a bacterial infection but you do not have enough resources to confirm the diagnosis. It is a challenge because it leaves you in a dilemma of whether to prescribe antibiotics or antimalarial because there are no resources to help confirm the diagnosis. You might prescribe antimicrobial in a case which does not need antimicrobial prescription.’

‘Shall we continue with the challenges?’

‘That’s enough’

‘So if I quoted you very well, you mentioned that a patient would come and be tested MRT negative but the patient will still want to give them antimalarial?

‘Yes

‘You also mentioned that members of staff will have their own ideas. They want antimicrobial while the indications are not there?

‘Yes

‘You also mentioned another difficulty of confirmed diagnosis. You said there becomes a difficulty where the diagnosis is not ascertain but you will still want to prescribe antimicrobial?

‘Yes

**‘In your view, how do you describe the behaviour of your patient when you refuse to prescribe antimicrobials, both the antibiotics and the antimalarial? What is their attitude towards you?**

‘They would think that I’m not competent enough or that I do not want to prescribe them antimicrobial because I have other priorities apart from them. They would also think that there are other clinicians who can prescribe antimicrobials for them. In this case, much better than I am and I think they would even not come to me next time when they are sick again.’

**‘Do you have more attitudes?**

‘No.’

‘So the attitude where you refuse to prescribe antimicrobials you say they will think you are not competent?

‘Yes

‘They will think that you have a certain priority with the antimicrobials?

‘Yes

‘They will never come back to you and they will think the person who prescribed antimicrobials to them is more competent?

‘Yes

‘Okay, can I proceed?

‘Yes

**‘So, what communication skills are needed when you are prescribing antimicrobials? Both antibiotic and antimalarial?’**

‘One, we need to make sure that we are as open as possible with our patients and I have to make sure that I’m using more of the language which can be understood by the patient. I should make sure that I avoid using terminologies that cannot be understood by the patient. I should make sure that I explain to the patient clearly about their condition or I give them enough counselling on their disease, what is causing the disease they are having and what can be the right remedy against the disease they are having.

‘That’s all?

‘Yes

‘If I got you correct, you said the communication skills that are needed you have to be as open as possible to your patient?

‘Yes

‘Okay, you also mentioned that you have to use the language that can be understood by the patient, not using the medical terminology or the jargons?

‘Yes

‘You also mentioned that there has to be enough counselling to the patient?

‘Yes

‘Okay, you have also to mention the right medication that has to be given to the patient that is what you said?

‘Yes

‘Okay, can I proceed?

‘Yes

**‘How much time do you spend with each patient?**

‘It depends, I cannot say that I spend such time with all patients. It depends on the severity of the cases. Those cases which may need much of my time I would spend more that 20-30 minutes on one patient. Those which are not severe cases, I would spend 10 minutes with each patient.

**‘The time that you have mentioned, how does it affect antimicrobial prescription?**

‘For example, if I don’t spend enough time on a patient, I’m not going to crack or come up with signs and symptoms or the necessary things which can give me definitive diagnosis of the patient but if I spend more time with the patient, I am going to have more history of the patient and thorough physical examination and that is going to determine what kind of investigations I will do. That will make me come up with proper definitive diagnosis.’

‘Thank you. Can we proceed?

‘Yes.

**‘Would you described some of the guidelines that are used by clinicians during antimicrobial prescription? Both antibiotic and antimalarial?’**

‘Yes. Of course. In our healthy facility, we have clinicians who prescribe antimalarial to patients who do not have malaria or to patients who are having pains but the signs and symptoms are not consistent with bacterial infection and even lab investigations do not show anything to do with systemic bacterial infection. I would say that if there were good or standard guidelines for everyone to follow otherwise a lot of clinical officers usually prescribe antibiotics to those who are not having systemic bacterial infections and even those who are not having malaria, sometimes the patient will have malaria negative but they will choose to write positive and prescribe antimalarial.’

‘Thank you. So, what are some of the guidelines that can be used by clinicians when prescribing antimicrobials both antimalarial and antibiotics?’

‘So far, I would say that if a patient is to be prescribed antimicrobials, they should fulfil all criteria which require one to prescribe antimicrobials. For example, there must be signs and symptoms consistent with systemic bacterial infection and the lab investigations should confirm as well, as well as the examination. So one, if there are standard operating procedures pasted in all the wards as well as points where clinicians meet patients. Two, if the pharmacy can have a system where they would check if the patient result really is what is in the book. For example, if it is computerized and they can confirm the results that are in the book with the results that are in the lab that can also help to prevent unnecessary prescription of antimicrobials.’

‘So what are the guidelines you know that can be used by a clinician when they are prescribing antimicrobials?’

‘Can you explain on that?’

‘What I mean is, we have several guidelines so I’m looking for the names of the guidelines that can be used by clinicians as reference when they are prescribing antimicrobials.’

‘Okay, for example, Malawi Standard Treatment Guidelines (MSTG) which is used by clinicians and doctors. If every clinician is given that booklet, that can help otherwise, there are a very few who have that book and if they also have that soft copy and give to all the clinicians so that whenever they are stuck somewhere, they can refer to the guidelines and we also have those papers which can be pasted on the walls in the wards and departments.’

‘Thank you. Any more guidelines you know?

‘So far, that’s all.

**‘In your own understanding, have you ever heard of bacterial resistance?**

‘Yes.

**‘In your own words, what is it?**

‘Bacterial resistance is when one is exposed to antibacterial treatment or antimicrobial when he’s not necessarily having that infection or when he had that infection but he was just given for a short period of time as partial treatment. So what happens is that the bacteria will produce or develop another mechanism that will withstand that antimicrobial when it comes back in the body.’

**‘Thank you. Do we have an example of antimicrobials or antimalarial that have developed resistance in the body?’**

‘Yes, we have. The antimalarial we have fansidar and the antimicrobials we have chloramphenicol, just to mention a few.’

**‘What is meant by antimicrobial resistance?’**

‘Antimicrobial resistance means the causative organisms, the bacteria has developed a mechanism or a resistance to that antimicrobial which means you might give antimicrobial which previously could work or the bacteria could respond or could be sensitive to that antimicrobial but now in the later stage or after a certain period of time the bacteria will develop another mechanism against that antimicrobial.’

**‘What are the factors that lead to antimicrobial resistance for the antibiotics and the antimalarial?**

‘As I said, one is partial treatment, two, and prescription of antimicrobial in cases which do not have that condition at all. Three, even poor compliance to the antimicrobial which patients usually receive. If they are being prescribed, actually they have that infection but if they are prescribed, now they do not adhere to the drug. Let’s say for example, they get a drug which is supposed to be taken every 4 hours, then they choose to take it every 8 hours, that can be one of the factor.’

‘Any other factor?

‘That’s enough.

‘Okay. So if I have quoted you well you said that partial treatment. So who do this partial treatment? The clinicians or the patients?’

‘Partial treatment can be done by clinicians.’

‘You mentioned partial treatment, cases that do not have the condition to be with regular drugs and you also mentioned poor compliance?

‘Yes

‘Can we proceed?

‘Yes

**‘So who is responsible to solve this problem?**

‘That problem is the responsibility of management at the health facility as well as personal responsibility of each and every clinician in the hospital.’

‘Okay. Thank you. There is more addition that you want to add from this interview?’

‘No. There is no anything to add on that.’

‘Thank you for participating in this study, your voice will be kept confidentially.’

‘Thank you.’

‘
