# Supplementary material for: Determinants of clinician and patient to prescription of antimicrobials: Case of Mulanje, Southern Malawi
Source: PLOS Glob Public Health. 2022 Nov 16;2(11):e0001274. doi: 10.1371/journal.pgph.0001274 (PMC10022363; doi:10.1371/journal.pgph.0001274)
Supplement: S8 Text — (DOCX) [file pgph.0001274.s009.docx]

**8.APPENDIX: 8, In depth interview with clinician number 8 on determinants of antimicrobial prescription at Mulanje District, Malawi.**

‘Good afternoon, Sir

‘Afternoon, Sir

‘I am Morris Chalusa, a Clinical Officer working with Mulanje District Hospital, I am also a student at College of Medicine, doing Master of Science in Health Sciences (Antimicrobial stewardship). I’m doing a study called determinants of decisions between clinicians and patients to prescribe antimicrobial: A Clinician Perspective. I have a questionnaire that I will use. I’m going to assure you that all the recording that will be recorded here will be kept confidential. The only people who can assess this is myself and my supervisor, Chiwoza Bandawe and the third person who is going to analyse this data. You are free not to mention your name in this study, you are also free to terminate anytime that you feel you are offended in the interview, you are also free not to answer any questions that you feel are not relevant. The results of the study will be presented to a handover meeting as part of part of the feedback. Thank you. Can we start?

‘Yes, we can proceed

**‘What is your role at this hospital?**

‘I’m a Clinical Officer

**‘Where do you conduct most of your work?**

‘Mostly in the OPD

**‘Do you prescribe antimicrobials? Both antibiotics and antimalarial?**

‘Yes, I do

**‘Which one do you prescribe most between antibiotics and antimalarial?**

‘Antibiotics

**‘Why do you think you do prescribe a lot of antibiotics?**

‘Mostly it’s because there are a lot of infections that come with patients

‘Can you specify the infections?

‘Most especially they come with coughs

**‘In average per day, how many times do you prescribe antimicrobials both antibiotics and antimalarial?**

‘On average it is more than 4 times per day

**‘Please share with me factors you know that influence antimicrobial prescription**

‘More especially, it is the availability of indications for that antibiotic’

‘Any patient factor?

‘It is also because sometimes they have tried other antibiotics so we try to use to use different ones

‘Any more patient factors that can influence you to prescribe antimicrobials?

‘Sometimes they also have some choices and they even force me or tell you what they want

‘Any more factors that influence antimicrobial prescription?

‘No. I have exhausted

‘so if I have quoted you well, you are saying patient factors that can influence you to prescribe antimicrobials you mentioned of indication, you also mentioned that maybe patient’s condition is not improving so you change to a different antimicrobial and you also mentioned patient’s choices. You mentioned that patients will tell you to prescribe antimicrobials.

‘Sometimes they say I do better when I take this, so yes

‘So you prescribe them the antibiotics?

‘We relate.

**‘Okay. When did you start prescribing antimicrobials?**

‘Since I came out of school in 2008

**‘What problems do you face during this period when you started prescribing antimicrobials?**

‘the problems that we are facing are that there is most of all scarcity of these antibiotics, sometimes patient is resistant to the antibiotics which requires us to switch to others and sometimes the patients themselves come already having something in their minds to tell you I want this. This is another problem

‘Any more problems?

‘Yes, the other problem I said is the scarcity of the drugs themselves and some other antibiotics are coming with side effects which are difficult to manage as well.

‘So in terms of problems you mentioned that scarcity of antibiotics is a problem to you, you mentioned also that some of the antibiotics are resistant, you also mentioned that some of the patients will already come with what they want in mind. You also mentioned that some of the side effects of the antibiotics are difficult to manage?

‘Sure

**‘Do you have specific antibiotics that you have got side effects that are hard to manage?**

‘Yes. As of now we talk of chloramphenicol which causes diarrhoea in patients and sometimes anaemia.

‘Do you have any problems related to when you started prescribing antibiotics apart from these you have mentioned?

‘No other problem.

**‘Can you explain to me patient’s beliefs about antimicrobials? What do your patients believe about antimicrobials?**

‘Mainly, patients believe that when they take the most strongest antibiotics, then they will be cured and others sometimes believe that they should take antibiotics regularly just to clean their bodies. These are some of the beliefs.

‘Okay. Any more beliefs?

‘No.

‘So you mentioned that patients believe that if they take the strongest antibiotic they will be cured and you also mentioned that they want to take antibiotics regularly?

‘Yes, even though they are not sick

**‘Do you have anything to add or we should proceed?**

‘We should proceed.

**‘What challenges do you encounter when you are prescribing antibiotics and antimalarial?**

‘The challenges. One can be the scarcity of the antibiotics or the drugs themselves. Two, it is the already prescription that clients can have in their minds, they want to tell you what they want. The other thing is that we are forced to write antibiotics to the patient depending on what is available.’

‘Any more challenges?

‘No, that is all.

‘So, in terms of challenges, you have mentioned that scarcity of antibiotics is a problem also that some patients come already having in mind what you should prescribe for them and that you are forced to prescribe antibiotics that are available?

‘Yes.

**‘In your view, how do you describe the attitude of your patients when you refuse to prescribe antimicrobials?**

‘When we refuse to prescribe them with antimicrobials, they take us as those who do not know their work, we are not called good doctors because we are refusing them what they want so their attitude towards us is always bad.

‘Any more attitude?

‘Sometimes they even think of switching clinicians. They go to another clinician and whatever they get from you, they can even throw away.

‘Any more attitude?

‘That’s all.

‘So if I have quoted you well, you said that the attitude of patients when you refuse to prescribe them antibiotics, they think you are not a good clinician, they throw away what you have prescribed and you also mentioned

‘They switch a clinician.

‘Okay. Thank you. Can we proceed?

‘Yes.

**‘What communication skills are needed when you are prescribing antimicrobials to patients?**

‘You need to explain their condition and why we are giving them the antibiotics. They need to understand why we are giving them that specific antibiotic.

‘Any more communication skills?

‘The other explanation is that if they do not feel good in the prescribed time, they can come again for review and we also advise them to take their drugs regularly and if they are supposed to take them with meals, we tell them.

‘Okay. Any more communication skill?

‘The other skill we do is to provide them some of the possible side effects that they can face and when to come back if it is possible.

‘There is any more?

‘No.

‘So in terms of communication skills you said that we should explain the condition to the patient, to explain why they are taking the antibiotics, they should also come for review when they have got problems, they should take the medications regularly and also provide the information on the side effects as well?

‘Yes.

‘Can we proceed?

‘Yes.

**‘How much time do you spend with your patient when you are prescribing the antimicrobials?**

‘Most of the times, it is less than 10 minutes because of workload.

‘So how does it affect your job as a clinician?

‘This affects us because we do not do much as required to the patient because we are supposed to have enough time like 15 minutes with the patient so that we can have head to toe examination and thorough investigations plus full explanation of the treatment so that the patient can be satisfied when going home.

**‘How does it affect antimicrobial prescription?**

‘If you do not have much time to do investigations and examinations, then you can even miss the diagnosis and give wrong antibiotics?

**‘Can you describe some of the guidelines used during prescription of antimicrobials; antibiotics and antimalarial by the clinicians?**

‘Our guidelines depend on the symptoms of which we can talk of fever and the associated issues like description of a sepsis.

‘So what are the guidelines that are being used for prescription of antibiotics?

‘We can talk of glam positive or glam negative depending on the kind of bacteria itself, how sensitive it is to a particular drug. When we talk of malaria, it depends on the severity.

‘Have you ever heard of antibiotic resistance?

‘Yes.

**‘What is antibiotic resistance?**

‘The resistance is when we give the same kind of antibiotic to the patient without getting the intended results simply because there is mutation or change of bacteria or whatever change of the bacteria which can do with the antibiotic itself.

**‘What is meant by antimicrobial resistance?**

‘This means lack of effect even if we give the antibiotic.

**‘Okay. In your understanding, can you describe some of the factors that lead to antimicrobial resistance?**

‘Yes, this can be irrational prescribing and much availability of these antibiotics to the vendors who just sell without normal prescription and sometimes unfinished drug courses.

‘There is any more?

‘That’s all.

**‘So whose responsibility it is to prevent antibiotic resistance?**

‘I n the beginning, it is ourselves, we need to be prescribing antibiotics rationally and management as well has to control the availability of these antibiotics outside the hospitals.

**‘Okay, do you have anything to add on the recording?**

‘I will just appreciate. Thank you for including me in your case and I think my contribution will change in the way these antibiotics are being used.’

‘Thank you for participating in the study, your availability will contribute a lot to the policy changing of how we can use the availability of resources in terms of distributing antibiotics. Thank you for participating.’

‘You are welcome.’
