# Supplementary material for: Determinants of clinician and patient to prescription of antimicrobials: Case of Mulanje, Southern Malawi
Source: PLOS Glob Public Health. 2022 Nov 16;2(11):e0001274. doi: 10.1371/journal.pgph.0001274 (PMC10022363; doi:10.1371/journal.pgph.0001274)
Supplement: S9 Text — (DOCX) [file pgph.0001274.s010.docx]

**9 APPENDIXES: 9, In-depth interview with clinician number 9, on determinants of antimicrobial prescription at in Mulanje District Malawi.**

‘Good Morning?

‘Good Morning.

‘How are you?

‘I am fine and how are you?

‘I am fine. I am Morris Chalusa, a Clinical Officer at Mulanje District Hospital and I am also a student at the University of Malawi, College of Medicine doing Master of Science in Health Sciences (Antimicrobial Stewardship). I’m doing a study called Determinants of decisions between clinicians and patients to prescribe antimicrobials. My study will look at factors that promote antibiotic resistance. We will have an interview for around 20-30 minutes. You are free not to mention your name in this study, you are also free to terminate anytime that you feel you are offended in the interview, you are also free not to answer any questions that you feel are irritable. The recordings will be kept safe in a secret place. The only people that will be able to access this interview are myself, Morris Chalusa, associate Professor Chiwoza Bandawe or Dr Felix Khuluza who are my supervisors and the third person is the one who is going to help me in the analysis of this data. Thank you. Can we proceed?

‘Yes, we can proceed.

**‘What’s your role as a health worker in this hospital?**

‘I am a Clinical Officer

‘Do you prescribe antimicrobials?

‘Yes, I do.

**‘Where do you conduct a majority of your work?**

‘In paediatric ward.

‘So you said you do prescribe antimicrobials? Both antibiotics and antimalarial?

‘Yes, I do.

**‘Which one do you prescribe most between the antibiotics and antimalarial?**

‘Antibiotics.

**‘Do you have a reason for prescribing more antibiotics?**

‘Yes. Some of the reasons are that I rule out if one has malaria by doing malaria test and if they are negative, if there is presence of doing full blood count, if I do and I find out that the patient has infection then it’s when I prescribe for the antibiotics.

**‘In average per day, how many times do you prescribe the antimicrobials? Both antibiotics and antimalarial?**

‘Let’s say 5

**‘Would share with me patient factors that you know that influence antimicrobial prescription?**

‘The signs and symptoms. When one is presenting them. Like when one is presenting fever, body pains

‘Anymore?

‘Generalized body pains (3:58)

‘Do you have any patient factors that will influence you?

‘No, there is nothing else.

‘So if I quoted you well you mentioned that presentations can also influence you to prescribe antimicrobials?

‘Yes

‘And also the symptoms?

‘Yes

‘Should we proceed?

‘Yes, we can

**‘When did you start prescribing antimicrobials?**

‘More than 2 years.

**‘What problems do you face during this period when you started prescribing antimicrobials?**

‘Sometimes shortage of the antimicrobials. Sometimes patients do not respond to a specified antibiotic that you have prescribed. Those are the main ones.

‘So, in terms of the problems you have mentioned of shortage of antimicrobials and you have also talked of patients not responding to the antimicrobials?

‘Yes.

**‘Can you explain to me patients’ beliefs about antimicrobials?**

‘They believe that once they receive the antibiotics, they will get better quickly especially the injectable unlike the orals

**‘Any more belief?**

‘That it is the best medicine in the world.

‘By the best medicine, you mean the injectable or the antibiotic?

‘Both. The fact that they are antibiotics.

‘Any more belief?

‘You are the good clinician.

‘When you say you are the good clinician what do you mean? When you prescribe them the antibiotic? Or?

‘Yes, when you prescribe them that’s their belief. If you don’t they think that you didn’t assist them well.

‘Any more belief?

‘That one should be of antimalarial. Not prescribing them antimalarial when they have signs and symptoms of malaria, I think it is still falling on you are not a good clinician.

**‘Do you have any more beliefs from your patients?**

‘No.

‘So if I have quoted you well you said they believe that antibiotics are the best medicine in the world, they also believe that when you give them, they will be cured. They also believe that the injectable are the best ones than the orals?

‘Yes.

‘They also believe that if you don’t give them the antimicrobials, you are being a bad clinician and they also believe that you do not want to assist them?

‘Yes.

‘They also believe that if they are showing the signs and symptoms of malaria then you have to prescribe them with antimalarial even if the results are negative? That’s what you mean?

‘Yes.

‘Generally, since you started prescribing antimicrobials, there is a patient who came to you who you testes malarial diagnosis test which came out negative, did a full blood count was normal?

‘Yes.

**‘What are the challenges of antimicrobials prescription? Both antibiotics and antimalarial?**

‘Like the scenario that you have given me?

‘No. Any. That was just an example. You can base on this story or any challenge since you started prescribing antimicrobials.

‘The patient still needs to be prescribed despite your explanation on the findings. Like the scenario where you have given that what you found is malaria negative, you don’t prescribe antimalarial and if full blood count is normal you don’t prescribe antibiotics. So, like my case I give what I feel like analgesia and counselling so to the patient they feel like you haven’t assisted them. The challenge is that you are not supposed to prescribe that one.

Then challenge more

‘Or should we skip that one?

‘Yes.

**‘So in your view, how do you describe the attitude of the patients when you refuse to prescribe the antimicrobials? Both the antibiotics and antimalarial**

‘They feel you are not a good clinician and you haven’t assisted them.

‘Any attitude.

‘They are disappointed some sort of.

‘Any more attitude.

‘Some don’t feel satisfied with the explanations as to why we are refusing them to have the treatment they wanted.

**‘Okay. So, what communication skills are needed or do you use when you are prescribing antimicrobials to patients?**

‘Is it after I have done all the investigations and now I want to prescribe antimicrobials?

‘Yeah, or you can answer it that way.

‘On how they are supposed to take it and how long?

**‘I’m not sure. That is what I want to find out from you. Say you were interacting with a patient so you want to prescribe antimicrobials, what communication skills are needed?**

‘I think you need to communicate on the dosage; how to take it and for how long and if there are side effects. Those things need to be communicated.

‘Anymore?

‘And when to return to the hospital if there is no improvement.

‘That’s all?

‘Yes.

‘Thank you. So, in terms of the communication skills you need to make on the dosage you mentioned how to take it in terms of the frequency, duration, when to return and the side effects?

‘Yes.

‘Okay.

‘The name of the antibiotic too.

‘Okay. Can we proceed?

**‘How much time do you spend with each patient?**

‘A minimum of 10-15 minutes.

**‘Why do you spend so much time with the patient?**

‘Let me go back. Is it during the prescription time? Or?

‘Any time you spend with your patient.

‘I spend at least 10-15 minutes because they need to understand how these medications work, if there are any problems, they need to know as well and because we also need to talk of the issues like resistance if they do not complete the dosages because some can just take for a few days and once they feel better, they stop taking the medications so we need to explain to them so they make an informed decision.

**‘Can you describe some of the guidelines that are used during prescription of antimicrobials? Both antibiotics and antimalarial per patient?**

‘The MSTGs

‘What is the MSTG?

‘The Malawi Standard Treatment Guidelines.

‘Okay.

‘The malaria guidelines and there’s the bluebook paediatric handbook, the clinical handbook, the bluebook.

‘Any more guidelines?

‘No.

‘Okay. If I have quoted you well you mentioned the Malawi Standard Guideline Treatment, the malaria guidelines, the clinical bluebook and the paediatric handbook?

‘Yes

**‘Have you ever heard of bacterial resistance?**

‘Yes

**‘In your own words, what is it?**

‘It is when the bacteria that cause diseases is not responding to the antimicrobials.

‘In your own words, what are the examples of antibiotics or antimalarial that are resistant?

‘Doxycycline and erythromycin, quinine, ceftriaxone and penicillin.

**‘In your own words, can you define what is meant by antimicrobial resistance?**

‘It means that the antimicrobials are not working on the specific bacteria, that is, in terms of curing the patient with that kind of antimicrobials. One does not respond to treatment.

**‘Can you describe factors that lead to antimicrobial resistance? Both antibiotics and antimalarial?**

‘Under dosage. Giving to patients when they are not supposed to receive those antimicrobials or antimalarial. The other could be those receiving from these other clinics unregistered because they just give anyhow.

‘Anymore?

‘No.

‘So, in terms of factors that lead to antimicrobial resistance you mentioned of under dosage, you also mentioned prescribing to patients who are not supposed to receive antimicrobials, you also mentioned buying drugs from those that are not registered?

‘One can also be prescribed the right antibiotics but if they are not taking it that can also be a contributing factor. I think it is falling on under dosage.

**‘Who is responsible to solve this problem?**

‘I think it is both the clinician and the patient.

‘Why?

‘Because if the patient is aware of where to receive the right treatment and then if he is prescribed with the right treatment and decides not to complete the treatment and the clinicians have to follow the guidelines and they should prescribe the antimicrobials where necessary not just to please the patient. So, it is two way to me.

‘Thank you. Do you have any addition on what we have been discussing?

‘No.

‘Okay. Thank you very much for participating in this study.
