# Supplementary material for: Determinants of clinician and patient to prescription of antimicrobials: Case of Mulanje, Southern Malawi
Source: PLOS Glob Public Health. 2022 Nov 16;2(11):e0001274. doi: 10.1371/journal.pgph.0001274 (PMC10022363; doi:10.1371/journal.pgph.0001274)
Supplement: S10 Text — (DOCX) [file pgph.0001274.s011.docx]

**10. APPENDIXES:10, In-depth interview with clinicians’ number 10 on determinants of antimicrobial prescription at Mulanje District, Malawi.**

**IN-DEPTH INTERVIEW:10**

I am Morris Chalusa. I am a clinical officer working with Mulanje hospital. I am also a student at college of medicine, doing Masters of Science (Antimicrobial stewardship). As part of my academic, one of the recommendation is to do research so I decided that I will do my research at Mulanje district hospital and Mulanje mission hospital. So I have also identified you as my participant. We will do our conversation in about 20 to 40 minutes. Questions that you see that are not appropriate to you, you are free not to answer them. If you want to stop the interview at any time you are free to tell me, we can stop. Our conversation will be kept secret. You are also free not to mention your name, we’ll just start asking you question, thank you

**-**All right, go ahead

**What’s your role at this hospital?**

**-**Am a clinical officer

**As a clinical officer where do you conduct much of your work?**

-In pediatric ward

**Thank you. Do you prescribe antimicrobial both antibiotics and antimalarial?**

-Yes

**Which ones do you prescribe most?**

**-**The **Benzylpenicillne, the gentamicin, gentamicin,** the **cephalosporins like ceftriaxone.** These are the most common drugs I use and sometimes we use **Artersunate, for malaria, LA, Lumefantrine - Artemether,** sure.

**Why do you think you do prescribe these most, the antimalarial, antibiotic as you have mentioned? Why do you prescribe them most?**

**-T**hey are most common in our hospitals

**On average per day how many do you think you prescribe most?**

-More than 4 times

**Which are the most prescribed ones? The anti-malaria and the antibiotic**

- **Lumefantrine - Artemether**, **atertusane based penicillin, and gentamicin**.

**Share me what you know about patient factor that influence microbial prescription? What are the patient factors that influence you to prescribe antibiotic and anti-malaria?**

-There are some patients when they come to the ward they have a belief that they can be healed by a particular drug. To them they believe that that drug is good to them. The other factor is that they have I can say an infection that whatever you can give to them is okay without asking any question.

Any belief?

-I don’t think there is another belief

Okay. So you have mentioned that patients normally believe that the particular drug that you are prescribing will help them when they come to the hospital, they also feel that when you prescribe them the drug it will be okay for them.

**When did you start prescribing antimicrobial both antibiotics and antimalaria?**

-2012

**What problems did you face during this period when you started prescribing antimicrobial? Both antibiotics and anti-malaria**

-Sometimes its shortages or out of stocks of drugs. There are some drugs which like the antimicrobials I mean ant-TBs, they are difficult to prescribe in the ward without a proper training. So lack of training. I think as of now that’s what I can say.

**So you mentioned shortage of drugs as a challenge**

-And there are some drugs which needs training for one to prescribe like and for example the TB drugs like say drug resistant TB, you cannot just wake up and prescribe. You need to be trained. Once you are trained you will be familiar with combination of the drugs

You mentioned shortage of drugs and sometimes they are difficult to prescribe which means they need trainings, you mentioned there is lack of trainings so the other **TB** drug resistance they need to be prescribed when you have gone to training. Any problem so far? Any challenge when you started prescribing antimicrobials?

-No

**Okay. Explain to me your thoughts regarding patient factors and beliefs about antimicrobials? What do you think are patient factors and beliefs about antimicrobials? What do think patient believes about antimicrobials, antibiotics and antimalarial? What do they think about it?**

-The patients?

Yeah

-Sometimes when the patient is not doing well, when they come to the hospital they believe that they would be cured and when they are not cured they believe you are giving the wrong drug

Any belief?

-Sometimes patients believe that once you start giving them a drug they will cured instantly

Any belief?

-I think that’s all

So you mentioned that patient believe that when have been prescribed a drug they will be cured instantly, patients also believe that when they are not doing better, they not cured then they are given a wrong drug. So what happens when they feel you have been giving them the wrong drug? What do they do?

-Some patients they may ask you that they should be transferred to another hospital say the paid ones, mission.

Anything to add on this?

-No

**What challenges do you encounter when you prescribe antimicrobials? Antibiotics and antimalarial**

-Challenge number one as I have already said is shortage of drug. The other challenge is about the storage facilities like in the ward. You may find that you have maybe 5 patients and you have prescribed the drugs for all the patients. If you collect the drugs for the, for the nurses to store the drugs it’s difficult and sometimes they maybe mixing up for other patients

Any challenge?

-No

So you mentioned shortage of drugs and storage problems as challenges you have encountered so far.

**In your view how do you describe the attitude of your patients when you refuse to prescribe antimicrobials? You say the patient is sick and you have talked them that you will not give them thus medication because of A, B, C, D. What’s the attitude?**

-When you prescribe the antimicrobials to patients they believe that their problem has been solved and they feel good.

So if you refuse, if they say okay, so you say **Malaria diagnostics test is Negative**. But normally when **MRDTs** negative but when I take antimalarial or antibiotics I feel good;

**What do you do?**

-Counselling. We need to counsel, I do counselling though it’s difficult for these patients to understand but counselling is important. So we counsel them on the dangers of taking antimalarial if **MRTDs** is negative

**So if the counselling has been done and your patient shows unsatisfactory what do your patients?**

-Like in my case we even consult with seniors that they should sit down with the patient for his understanding

**What communication skills are needed when you are prescribing antimicrobials?**

What communication skills are needed when you are prescribing these antimicrobials to patients?

-The health worker and patient interaction

When you have the patient there and you are prescribing here so what communication skills are needed?

-I don’t understand

So let’s make an example. You are my patient or I am your patient, you are my clinician. So you are prescribing antimicrobials what skills are needed when you are prescribing antimicrobials? Both antibiotics and antimalarial

-Counselling, you should have the skill of counselling that they should understand what you are giving

Any skill?

-I don’t know if that would be the skill but examination. Examination the patient that makes the patient feels like he has been helped.

Any skill?

-So far no

**How much time do you spend with each patient when you are prescribing antimicrobials?**

-On average, 15 minutes

**Can you describe some of the guidelines that are used during prescription of antimicrobials for clinicians?**

-The common one we have MSTG; Malawi Standard Treatment Guideline. That’s the common one which we are using

Any other guidelines?

-We have the TB manuals, the ART manual, that one is general. The pediatric handbooks, the blue manual, the malaria guidelines

Any?

-That’s all

So you have mentioned Malawi Standard Treatment Guideline, the TB manual, ART, Pediatric handbook. Which pediatric handbook is this?

-The most common one we are using Kazembe peadiatric book and we have a white book from Queens for peadiatrics

So the pediatric handbook for Kazembe, the white book from Queens, the TB manual.

**Have you ever heard of antibiotic resistance?**

-Yes

What is it?

-They are some conditions which when we prescribe drug they don’t respond to that particular drug so we say that there is a resistance to that condition pertaining to the drug given.

So you are saying antibiotic resistance is a type of resistance whereby you are giving a drug and that drug is not responding to that particular condition. Then you say that is resistance.

-Sure

**So what is meant by antimicrobial resistance?**

-In simple terms we can say a condition which is not responding to a particular drug

So you are saying a condition which is not responding to a particular drug.

**Can you describe factors that leads to antimicrobic resistance? Both antibiotic and antimalarial**

-Unnecessary prescription of antibiotics and antimicrobials and sometimes patients they believe that there is a drug for any condition. That means once they come to the hospital they want to move out with the drugs. So coming to the hospital to them you need to give them the drug then it also contributing to drug resistance

Okay, so you also mentioned patients believe that there is drug for any condition. This also contribute to antibiotic resistance.

Any factors?

-As of now, no

**Whose responsibilities is to resolve the problem of antimicrobial resistance?**

-Both

Who?

-The clinicians and the patient

Why?

-As I have already said the patient should understand that there is no, we cannot give drugs as they wish, but according to their condition. The same applies to the clinicians we should not prescribe drugs for the sake of prescribing. We need to understand how they work

So you are saying both clinician and patient, the patient should understand how the drugs works and as you as technician they should not just prescribe medication, the clinician should also understand how the drugs works. That’s what you have said

-Sure

Any addition to what we have discussed?

-No

No. Thanks.

Your contribution is very important and we will keep this recording on the safe side.
