# Supplementary material for: Determinants of clinician and patient to prescription of antimicrobials: Case of Mulanje, Southern Malawi
Source: PLOS Glob Public Health. 2022 Nov 16;2(11):e0001274. doi: 10.1371/journal.pgph.0001274 (PMC10022363; doi:10.1371/journal.pgph.0001274)
Supplement: S11 Text — (DOCX) [file pgph.0001274.s012.docx]

**11. APPENDIXES:11, In-depth Interview with Clinician number 11, on determinants of antimicrobial prescription at Mulanje District, Malawi.**

**IN-DEPTH INTERVIEW: 11**

Good morning

-Good morning to you too

I am Morris Chalusa. I am a clinical officer working with Mulanje hospital. I am also a student at college of medicine, doing masters of Science (Antimicrobial stewardship). As part of my academic, one of the recommendation is to do a research so I decided that I will do my research at Mulanje district hospital and Mulanje mission hospital. So I have also identified you as my participant. We will draw our conversation in about 20 to 40 minutes. Questions that you see that are not appropriate to you, you are free not to answer them. If you want to stop the interview at any time you are free to tell me, we can stop. Our conversation will be kept secret. You are also free not to mention your name, we’ll just start asking you question, thank you

**What is your role?**

-I am a medical assistant

**Medical assistant, where do you conduct the majority of your actual work?**

-Outpatient department

**Do you prescribe antimicrobials?**

-Yes

**Which ones do you prescribe most?**

-Mostly, **Co-trimoxazole** antibiotics, antimalarial and we have so many when we have so confirmed cases we have to prescribe

**Thank you. Why do you think you prescribe most of these antibiotics?**

-Mostly we are not sure of the infection we are treating. We just think when we cover them with antibiotics we are covering them, yeah.

**On average per day how many times to prescribe antimicrobials?**

-More than 10 per day, more than that.

**Between these two; antimicrobials and antimalarial which ones do you prescribe most?**

-Antibiotics

**Share me what you know about patient factors that influence antimicrobials prescription?**

-Inconsistence in the time of taking drugs. When they have been prescribed to taking either twice or three times days they might take longer or fewer time.

Any factors that influence you to be prescribe antimicrobials to patient?

-Sometimes these clients are related to some people that they are already directed to get some drugs when we advise them to go with drugs they prefer going to the next clinics just to get drugs

**Any factors that influence you to prescribe antimicrobials to patient?**

-Just to get rid some of them. Some they are frequently come to the hospital. They are not sure of what they are doing so to get rid of them we just maybe prescribes **Co-trimoxazole.**

**Any factors that influence you? You can mention as much as possible**

-Favour, buying favours. Sometimes we do it so people should see yeah

Okay, any?

-Lack of relevant antibiotics. We may need some **gentamycin** which is mostly lacking so we just go to the **Co-trimoxazole.**

Any factor?

-No

Okay, so you have mentioned points that makes you prescribe antimicrobials, relevant to people who direct them to you so that you should prescribe antimicrobials. Some of them you want to get rid of them because they usually come frequently to the hospital, you also mentioned buying favours to them, also mentioned of lack of antibiotics so you want to prescribe the same antibiotics, that’s what you are trying to say

-Yes

**Okay. When did you start prescribing antimicrobials?**

-2010

**What problems did you face during this period when you started prescribing antimicrobials?**

-Client understanding. They don’t understand when you give them drugs to take them maybe in 7 days. In less than that we are coming back we want more drugs. We explain to them, they don’t feel like satisfied or they change; they move from you they go to someone.

Okay, any problem?

-Lack of relevant antibiotics or drugs that you want to supply to clients, yes. We have no choice sometimes that we would only have that type of drug which is not supposed to be given in that condition (disease) that you feel the patient is in need of drugs.

So you have mentioned of client who don’t understand, patient also run away from you and going to other clinician. Is there other problem?

-Mostly it’s…

**Explain to me your thoughts in regard to patient factors and believes towards antimicrobials. What do you think are patient factors and beliefs about antimicrobials?**

- We have problems with patients because even if we don’t supply them with antibiotic here they go to the open markets and buy.

They don’t know how much provided they have managed to buy 2, 3 or more than that. This and proper advices, they don’t know which drug goes with what or which one is contradicting with this drug, they don’t know. It can be the hospital or it can be themselves.

Apart from those two you have mentioned, any beliefs?

-When they come to the hospital they believe to have collected drugs, no advice, because sometimes you can just give them advice but they don’t feel comfortable with advice only, they want something tangible to take home.

Any other belief?

-Patients would prefer to be given injections so when you prescribe just **orals drugs** yes he will leave the facility but he will go somewhere to be injected.

**Okay, thank you. What challenges do you encounter when you are prescribing antimicrobials and antimalarial?**

-Antimalarial, we don’t have much challenges because we have confirmed cases of malaria but on antibiotics we will need much of investigations much of that are not happening like Blood culture so you will just give on thinking.

Any other problems?

-Short supply of antibiotics especially like those **Chloramphenicol, Erythromycins**. The common found antibiotic is **Cotrimoxazole** think it’s because it’s also used in HIV

Any other problem?

Yes

-Commonly found in markets. It is also disturbing us because storage they don’t know. Expiring dates they don’t know. How they mention the names when buying we don’t know because we received somewhere people using TB drugs for healing wounds, applying on wounds or drinking as cough, a simple cough. Drinking maybe for 2, 3 days. The name they use there they don’t know.

So you have mentioned that the challenge that you are facing when prescribing antimicrobials especially antibiotic are short supply of antibiotics, the other problem is that of culture, you just treat patient empirically. Prescribing of some antibiotics being common in market because of some antibiotics like some that are used for TB treatment they are being used for treatment of cough. People will go and buy there, for healing wounds. Any other challenge? In terms of antibiotics.

-No

**Okay. In your view how do you describe the attitude of your patients when you have refused to prescribe them antimicrobials? Let’s say patient has come**

-I understand

So you say that to the patient

-Just go and take water

So how do they behave?

-A little number of patients understand when you explain to them what it is means taking a lot of drugs but some they don’t want to see you or meet you again. They would prefer to meet someone and they will not come back to you. But there are some who are understanding.

**Anymore attitude?**

-Some lose trust as I said. Some they want to come and learn more what you said about drugs. Sometimes we tell them such, such Drugs can cause these, can cause resistance.

-So with the attitude you have mentioned some will run away from you, some will lose trust, some will come back and want to learn more.

**So when you are prescribing antimicrobials what are the communication skills you need as a clinician?**

-Okay, first we need to explain why we are giving the drug, frequency, the root of taking the drug and duration. And they should not share with someone else because it’s only for him or them that have attended the service and the drug is prescribed only for him. I think on the problems people still share drugs somewhere behind.

So we are talking about communications that you need when prescribing drugs. So you have mentioned you need to explain the root, duration, not to share drugs for them, also frequency. Any other communication skills that is needed?

-First and foremost you need the patient to understand. You should build a good relationship between you and the patient and make sure when you have given the drug they should come back for feedback. Maybe they have improved or they have not. And you can see what to do by then some people will come and you gave me this drug and I have noted I have improved; give me some more. They will need some more because it has done well to them. But some will just change from one clinician to the other. And they will change the profile so that no one will see the past history for them. And we will give them good drugs at every visit.

So you mentioned that they should understand why you are giving them that drug, you have also to make sure that there is a good relationship between you and patient. You have also to provide information that they should come back and give you feedback how they have responded. Any skill that you want to add? You can mention as much as possible.

-I think the way you present yourself to the client will also talk whether to have trust, to lose. Yes, the tone of talking to the patients will also matter to the response of treatment.

So the way you present you to the patient will also help for the patient to understand, that’s what you are trying to say, you also mentioned the tone, it matters for a patient to understand. Is there anything?

-No I think I have taken by a little surprised so some…

**Okay. How much time do you spend with patient?**

-It depends on number of patients you have. When we have like 50 per day we’ll take time; 5, 7, minutes but when we have so many less than 3 minutes per patient.

**In terms of the guidelines. Would you describe the guidelines that are used in antimicrobial prescription?**

-There are some drugs that cannot be combined when prescribing like, for example, when you are giving someone LA you don’t give like **Metronidazole, Albendazole**, you correlate the ….the antibiotics and other drugs. And other antibiotics are not to be taken with beer as for those who take beer. And also other antibiotics they are not taken in pregnancy.

**So, I was looking at the guidelines that you are using during..**

-Yes the Malawi standard treatment guideline

MSTG

-We will always refer to them

Any other?

-HIV new guidelines, the TB/HIV they are related, the STI guidelines, the malaria control program, we have the guidelines.

So you mentioned the Malawi standard treatment guideline, TB guidelines, sexually transmitted infections guidelines. You also mentioned HIV guidelines, the malaria guideline. Thank you. Have you heard of antibiotic resistance?

-Yes

**What is it? What is meant by antibiotic resistance?**

-Failure to treat by antibiotics to certain infections that used to heal with the same drug.

Do you have examples of antibiotic resistance?

-Yes. I can see **Penicillin’s** are not working to some people.

Any?

-The TB drugs. For the development of **Mult Drugs resistant’s (MDR) TB** because there is resistance of drugs to some of these …

**So in your own words what is meant by antimicrobial resistance?**

-It’s failure to be successful in treatment of the same infection that used to be treated by the same drug because of change of, is it mode yeah

**Can you describe factors that leads to antimicrobial resistance? Both antibiotic and antimalarial**

-Improper use by us prescribers. We still have people that prescribe antimalarial when the result is negative just in preference or culturally believing that any fever is malaria. We have problems with the patient themselves that you’ve prescribed them no antimalarial they go and buy. They access them from elsewhere while they don’t have malaria parasites. Antibiotics, the same thing. They buy maybe for two days, three days yet they are supposed to take maybe for seven days or more. Frequency, some clients don’t know. They just take drugs any time maybe once a day for the drug they are supposed to be taken three times a day. So there a lot of factors that can cause resistance. Some people they can take a different antibiotic today for example **Amoxicillin**, today tomorrow they are taking **Co-Trimoxazole**, or the other day they taking **gentamycin** the same body for the same infection, I don’t think the drug can work.

Anymore?

**So, whose responsibilities is to resolve the problem?**

-Us and the clients

**Why do you think us and clients?**

-Us as providers we have to give more light as why we gave that antibiotic. Whenever we don’t have the supply we can explain we don’t have so that they can access, they can buy on themselves. We can just prescribe for them. And they should take them the way we want them to take so that is its too good for their own body for successful treatment and also to avoid resistance. Us as health workers sometimes we collide them ourselves with each other. My understanding won’t be your understanding at the same time, the medicine is dynamic. Sometimes there are people that are stuck to the old, old guideline, some are sophisticated who go on nets so there are different views of managing the patients. We need to work together. We need some interventions of revising on how we do the job.

That’s the end of our interview. Do you have anything to add?

-No
