# Supplementary material for: Determinants of clinician and patient to prescription of antimicrobials: Case of Mulanje, Southern Malawi
Source: PLOS Glob Public Health. 2022 Nov 16;2(11):e0001274. doi: 10.1371/journal.pgph.0001274 (PMC10022363; doi:10.1371/journal.pgph.0001274)
Supplement: S12 Text — (DOCX) [file pgph.0001274.s013.docx]

**Figure 12 APPENDIXES:12, with Clinician number 12 on determinants of antimicrobial prescription at in Mulanje District Malawi.**

**IN-DEPTH INTERVIEW:12**

**Morris:** Good morning sir?

**Participant:** good morning

I am Morris Chalusa. I am a clinical officer working for Mulanje hospital. I am also a student at college of medicine, doing masters of Science (Antimicrobial stewardship). As part of my academic, one of the recommendation is to do a research so I decided that I will do my research at Mulanje district hospital and Mulanje mission hospital. So I have also identified you as my participant. We will draw our conversation in about 20 to 40 minutes. Questions that you see that are not appropriate to you, you are free not to answer them. If you want to stop the interview at any time you are free to tell me, we can stop. Our conversation will be kept secret and you are unable to find these interviews. You are also free not to mention your name, we’ll just start asking you question, thank you

**What’s your role at this hospital?**

-Am a clinical officer

**As a clinical officer where do you conduct much of your work?**

-In male ward

**Do you prescribe antimicrobials?**

-Yes

**In average per day, how many times do you prescribe antimicrobials?**

-It depends with the how many patients I have that I feel might need antimicrobials. I can’t figure out how many times. Sometimes it could be, a day would go by without prescribing antimicrobials. Maybe because most of the cases that we receive are from the Out Patient Department (OPD). So it might happen that in the OPD they have already prescribed so you just continue with that. Unless maybe if you are doing other investigations in the ward and then you find that there is a need for antimicrobials.

**So, suppose you have been allocated to OPD how many times do you think you can prescribe antimicrobials?**

-More than four times

**Share me what you know about patient factors that influences antimicrobials?**

-Some patients you may find that maybe they are used to some medications in preference of the other so they might say I do well with this kind of medication and these other medications I don’t find them comfortable to use maybe I find that I vomit after taking them. Sometimes it might be true but most of the times they just prefer a certain kind of antimicrobial to the other. And also maybe in their mind they have the belief that when they come to the hospital, whenever they are going back home for them to feel that they have been helped they at least have to have some medications.

So maybe those are the kind of things that prompt some to prescribe antimicrobials.

So you are saying that sometimes they are used to that particular antimicrobial. The other reason would be they normally used that at home, they always want to stay with the antimicrobial at home. **So basically which kind of antimicrobial do these patients requests? We have the antibiotics, antimalarial**

-Well, they can range from antimalarial to antibiotics. Mostly maybe people may want antimalarial at home, just to keep. So that whenever they feel headache they always complain its malaria, I have some LA I have to get it.

**When did you start prescribing antimicrobials?**

-I think in 2015 when I started doing clinical attachments. When I was a student.

**So what problems did you face during this period when you started prescribing antimicrobials?**

-Sometimes it would be the choice of antimicrobials that are there depending on the condition of the patient. Your choices maybe out of stock in a particular pharmacy and that can affect your prescription as well. And the other thing is, you are not quite sure what you are treating. So you just prescribe but then you are not really sure like blinded. Treating blindly

So you are saying that you are not quite sure what you are treating. Choice, the one you are prescribing is not available, you go to the alternative. So, alternative might be, the broad spectrum or the narrow spectrum.

-The alternative might be the narrow spectrum. Maybe you were targeting, you really wanted the broad spectrum but is not available at that particular time. You are forced to do the narrow spectrum.

**What do you think are the patient’s beliefs about antimicrobials?**

-I think they feel that for them to get well then they have to take a certain type of antimicrobial. Whether you find that the malaria test is negative then they still have the feeling that for them to get well they have to take antimalarial. Or for them they just have a viral infection like cough or whatever or just a flu. They believe that for them to get well they have to take antibiotic like **amoxicillin** or what.

So you are saying that patient believe that when they take antimalarial they will feel better despite the MRDTs is negative. Or either they have got another type of infection, when they take antibiotic **amoxicillin** they will get better.so you still prescribe them with antibiotics?

-No, you try to reason with them that it’s not like all times that they have to take medications. Some of the infection will just go on their own. And there could be other infections maybe different from what they think.

**What are the challenges do you encounter when you prescribe antimicrobial? Basically, antibiotic and antimalarial. What are the challenges?**

-I think the other things that I have already said, you find that you want this other kind of antimicrobial but it’s not around, it’s not available. You are supposed to prescribe another which is less effective than the one you think. The other thing is that much as we don’t have much test like blood culture for you to see which specific antibiotic to prescribe against that kind of condition. You don’t have that chance. Maybe it’s expensive or maybe it’s not done in most laboratory. So you are just forced to prescribe either way.

So you are saying, your challenges are you want to give abroad spectrum but it’s not available. You give a less effective one. The other point is you want to confirm what’s causing the problem then the **blood culture** is not available then you just prescribe those antibiotics.

**In your view, how do you describe the attitude of your patients when you refuse to prescribe them antimicrobials?**

-Well, when you refuse they think maybe you are not a good clinician. You don’t really know your work or else you haven’t helped them. They would prefer to go to another clinician or else to go to another hospital where they feel they can be helped. They feel that you haven’t helped them.

So they feel like you haven’t helped them. They will go to another clinician or another hospital.

**What do you think are the communication skills needed when you are prescribing antimicrobials to patients?**

-Well, maybe we have to sensitize them that it’s not like every problem that they have that they have to receive an antimicrobial. Some of the illnesses can resolve on their own and some of the illnesses need proper investigations before prescribing the antimicrobial. You just have to have much time with them and explain to them the benefits, the disadvantages, the side effects of just taking medications not related to their condition.

So if I heard you well you were saying that communications skills needed; we need to sensitize the community, the importance and effects of antimicrobials and the benefits of antimicrobials and the dangers of antimicrobials if wrongly used.

**What are some of the guidelines that you use when prescribing antimicrobials?**

-Basically, you look at the condition and the way it has presented and then you like it’s not that severe according to criteria. I will take an example of malaria. For you to say this is really malaria there is a criteria that you use for you to categorize it so if you feel like it’s not uncomplicated you opt for maybe pure antimalarial. If you feel like maybe it’s maybe complicated then you opt for IV. And then same as antibiotics, sometimes based on full blood count the white cell count is so high you presume that there should be an underlying infection going on and when the white cell count is so high you opt for IV antibiotics. And if you feel that maybe it’s not that bad you can go for pure antibiotics. And if feel like the patient has been on pure antibiotics forty hours and there is no much improvement then you can then think of switching to IV.

**So in your ward, do you have the guidelines? That you are using. The guidelines that will say for this condition prescribe this type of drug.**

-The ones that I have seen are in our ward we haven’t seen like posted there. We, use maybe from literature, from books or from whatever we have in the books. What are the books saying? Like for this condition you have to give this.

Examples of the books?

-Like for the handbook, the blue book. The Malawi standard treatment guideline, HIV Guidelines book, TB manual

**Have you ever heard about bacteria resistance?**

-Yes

What is?

-The inability of the drug to kill or to stop the growth of the bacteria either because the bacteria itself has developed a certain layer in its cell that is resistant to the kind of drug which initially maybe in the past it was sensitive to it but then it becomes insensitive.

So you are saying antibacterial resistance is resistant to a certain type of bacteria whereby the drug is not working because that particular microorganism has developed a certain kind of layer which makes unable for that drug to work

-Yes

**Do you know some of the microorganisms especially bacteria that have developed resistance?**

-Maybe I can just brainstorm some; **cephalosporins, Penicillin,** even the HIV virus itself. In TB we talk of the drug resistance TB.

**In your words, what is meant by antimicrobial resistance?**

-I think it’s the ineffectiveness of the drug that has been caused by too much use of it such that the bacteria that used to be cured or stopped growing by that kind of drug has stopped doing that. Instead maybe whenever you treat it with particular drug it’s not responding to it. There is no response maybe regarding that particular drug.

**Can you mention a few microbial organisms that have developed resistance?**

-**Plasmodium falciparum,** initially we used to treat it with **quinine or Fansidar.** So it’s now stopped responding to such kind that’s why they introduced this LA and then some kind of **Salmonella**

Which group are the Salmonellas? Which type do they belong to? -The bacteria

Basically, we have the viruses, bacterial, the parasite. Any other group? Any other microbes that have developed resistance? You have mentioned the bacterial, you have mentioned the parasites which are the **falciparum** to be resistant to **S P. The Salmonella** which are also resistant to some of the antibiotic which we are using

-and also some **anaerobes.**

Like what?

-That’s what I know

Okay. The **Salmonellas**, do we have a type of drug are resistant to?

-Sometimes people could use like **Chloramphenicol** but sometimes it’s not that effective

**Just describe, what are the factors that leads to microbial resistance?**

-I can say abuse of antimicrobials. It might have led to that. Maybe the patients cannot complete the whole dosage and also maybe we mismanage them. Maybe we prescribe the wrong antibiotic to a certain particular condition.

Any other point you remember?

-That’s it.

So you have mentioned abuse of drugs from the patients, not completing their dosage. Maybe you have prescribed a wrong drug to the patient which is mismanagement.

-And also the wrong dosage.

**The wrong dosage as well. Who do you think has got the responsibility to resolve this problem? Antimicrobial Resistant**

-I think both the health care workers as well as the patients do have a role. They all do play a role towards this kind of resistance because if the clinician prescribes the wrong antibiotic it means he has contributed to that. And then if the patient cannot complete a certain dosage or maybe he is just taking the wrong medication. So we are all to blame in that.

Thank you, we have finished our interview. Do you have any addition to this question that I have asked you?

-No

Thank you

-You are welcome
