# Supplementary material for: Determinants of clinician and patient to prescription of antimicrobials: Case of Mulanje, Southern Malawi
Source: PLOS Glob Public Health. 2022 Nov 16;2(11):e0001274. doi: 10.1371/journal.pgph.0001274 (PMC10022363; doi:10.1371/journal.pgph.0001274)
Supplement: S13 Text — (DOCX) [file pgph.0001274.s014.docx]

**Figure 13 APPENDIXES:13, in-depth interviewer with Clinician number 13 on determinant of antimicrobial prescription at in Mulanje District Malawi.**

**Morris**: Good afternoon

**Participant:** Good afternoon

I am Morris Chalusa. I am a clinical officer working for Mulanje hospital. I am also a student at college of medicine, doing masters of Science (Antimicrobial stewardship). As part of my academic, one of the recommendation is to do a research so I decided that I will do my research at Mulanje district hospital and Mulanje mission hospital. So I have also identified you as my participant. We will draw our conversation in about 20 to 40 minutes. Questions that you see that are not appropriate to you, you are free not to answer them. If you want to stop the interview at any time you are free to tell me, we can stop. Our conversation will be kept secret and you are unable to find these interviews. You are also free not to mention your name, we’ll just start asking you question, thank you

**So what is your role at this district hospital?**

-I am a clinical officer

**Which ward do you perform majority of your work?**

-Female ward

**Do you prescribe antimicrobials?**

-Yes

**Which one do you prescribe most? Antibiotic and antimalarial**

-It depends with the way the patient has presented

**On average per day, how many times do you prescribe antimicrobial?**

-Four times per day

**Which one do you prescribe most, antimalarial and antibiotic in the ward setting?**

-Most time is antimalarial and if the patient was on **Artersunate** then we switches to LA so

**Share me patient factors that influence antimicrobial prescription. What I mean is factors that make you to prescribe antimicrobials from the patient**

-The way the patient is presenting

Presentation

-If the patient is not changing on the drug which we have given

Not changing condition

-And according to lab results

There is any other factor?

-I think I have forgotten some.

**When did you start prescribing antimicrobials?**

-2017

So you have mentioned that patient presentation is the factor that influences you to prescribe antimicrobials to patient. Based on lab results, do influence you to prescribe antimicrobials. When the patient is not changing it can also influence you to prescribe antimicrobials. And you also started prescribing antimicrobials in 2017.So you said based on results. What do you mean when you say based on results?

-Let’s say its full blood count, there might be less white blood cells, low red blood cells count. **Low blood reds or her white count,** we can give antibiotics.

What problems did you face during this period when you started prescribing antimicrobials?

-Like in our setting, Mulanje, antibiotics are scarce kind of.

Scarce of antibiotic. Any problem? That’s all?

-I think so

**Explain to me your thoughts regarding patient factors and beliefs of antimicrobials? What do you patient beliefs about antimicrobials? What does your patient believe about antimalarial and antibiotics? What do they believe?**

-Am not sure

**What challenge do you encounter when you prescribe antimicrobials? Such as antibiotics and antimalarial?**

-Sometimes you may prescribe the antibiotic which you think is the best and you find out that the hospital doesn’t have it.

Any other challenge?

-No

**In your view how do you describe the attitude of your patients when you refuse to prescribe antimicrobials? Some of the patient has come, you have done full blood count, and the results are in normal parameter. So you say, look your results are bla bla bla we will not prescribe any antibiotic. OR your patient has presented with symptoms, fever, you did Malaria Diagnostic Test it comes negative. You did full blood count, normal results. So you say go home it’s just a flu, drink a lot of fluids. How do they react to you?**

-They will say you are stingy as if the drugs are yours. The next time they are sick they will not come back to you. They will go to another clinician. Claim that you would not help them again.

So you mentioned you could be stingy as if the drugs is yours. They will not come back to you, they will go to another clinician.

**What communication skills are needed when you are prescribing antimicrobials to patients? What are the communications that are supposed to be there when you are prescribing antimicrobials? Antibiotic and antimalarial**

-I think we should be telling them what the drugs are. What the mode of action the drug is, the side effects of the drugs and how to take them.

So you say communications skills; we have to talk to them what the drugs are, what is the function of the drug that is taking? Mode of action of that drug, okay. The side effects of that particular drug that you have prescribed and how to take those drugs. When you say how to take drugs what do you mean?

-The frequency

In terms of what the drugs are; what do you mean?

-Is it the first line, the second line or the third line?

**How much time do you spend with each patient?**

-Like to me every time it’s 10 to 20 minutes

**Can you describe some of the guidelines that are used during antimicrobial prescription? Both anti malaria and antibiotics by clinicians**

-I have forgotten the name

Can we skip this one?

-Yes

**Have you ever heard of bacteria resistance?**

-Yes

In your own words, what is it?

-The bacterial are becoming resistant to the antibiotic you are giving

Okay. So what does it mean when you say there is resistance?

-The antibiotics are not effective enough to the bacterial

**Do you have examples of antibiotics that are not effective against bacterial?**

-As specific antibiotic to the specific bacterial?

Or just antibiotic that are not working on bacterial. Not specific towards bacterial but just a list of antibiotic that are not working

-We have **Ceftriaxone**, **Bactrim, Penicillin, and Amoxicillin,** that’s all that is.

So you have mentioned that some of the examples of antibiotics that are not working. You have mentioned of **Ceftriaxone,** penicillin, Bactrim (Cotrimoxazole).

**What is meant by antimicrobial resistance? In your own words**

-In my own words I can say it’s the bacterial are not responding to the antibiotics

**Can you describe factors that leads to antimicrobial resistance?**

-Giving antibiotics when it’s not necessary to give. Prescribing the strongest antibiotics instead of giving the first line or the second line

That’s all?

-I have forgotten some

So you have mentioned that unnecessary prescription of antibiotic, you have also mentioned prescribing strong antibiotic than the second one instead of the first line. I think you have mentioned two points.

**So whose responsibility is to resolve this problem regarding the resistance?**

-Us clinicians

Why us clinicians?

-We are the one who is dealing with the patient most of the time and we are the one who can decide to give the antibiotics to the patient.

Do you have anything to add?

- So far, no.

Thank you for participating in this study. Your conversation will be safe. It will not be aired to anyone else. It will be kept in a computer with password. It will not be shared to anyone.

-Thank you.
