# Supplementary material for: Determinants of clinician and patient to prescription of antimicrobials: Case of Mulanje, Southern Malawi
Source: PLOS Glob Public Health. 2022 Nov 16;2(11):e0001274. doi: 10.1371/journal.pgph.0001274 (PMC10022363; doi:10.1371/journal.pgph.0001274)
Supplement: S14 Text — (DOCX) [file pgph.0001274.s015.docx]

**14.APPENDIXES:14, with Clinician number 14 on determinants of antimicrobial prescription at Mulanje District Malawi.**

Good morning, sir?

-good morning

I am Morris Chalusa. I am a clinical officer working for Mulanje hospital. I am also a student at college of medicine, doing masters of Science (Antimicrobial stewardship). As part of my academic, one of the recommendation is to do a research so I decided that I will do my research at Mulanje district hospital and Mulanje mission hospital. So I have also identified you as my participant. We will draw our conversation in about 20 to 40 minutes. Questions that you see that are not appropriate to you, you are free not to answer them. If you want to stop the interview at any time you are free to tell me, we can stop. Our conversation will be kept secret and you are unable to find these interviews. You are also free not to mention your name, we’ll just start asking you question, thank you

**What is your role at this hospital?**

-I am a clinical officer

**As a clinical officer what do you do?**

-I do a lot of things; prescription, assisting patients, do a lot of procedures and doing surgeries

**Where do you conduct the majority of your work?**

-Mostly it starts in the OPD and the wards like notably I conduct clinics for such patients and the ward for those patients who are admitted. We keep on assessing and managing them. Basically, in the OPD and the wards

**Which ward particularly do you work?**

-Currently, am just floating. I don’t have a specific ward. So I normally get called especially in female male.

**Do you prescribe antimicrobials?**

-Yes I do

**Which one do you prescribe most?**

-Mostly, they are antimalarial and antibiotics

**Which do you think you do prescribe the most?**

-Let me start with the antibiotics. We see a lot of cases especially **Pneumonia**, bacterial skin infections that mostly warrants us to prescribe antibiotics. And for the antimalarial, for those who have malaria we assess and that they have malaria. We have tested and they have malaria and those are the patients you prescribe.

So you are saying that you normally receive a lot of patients including those that present bacterial infections so you prescribe medications depending on the condition or diagnosis they present.

You also said you prescribe antimalarial based on the MRTDs results. If they are positive you prescribe them. In average per day how many times do you prescribe antimicrobials?

-In average I might prescribe up to five apart from those in the wards because most of them when they are on antibiotic probably mostly we just continue of. Or at least could be five or more per day on average.

**So five or more in a day. Both antibiotics and antimalarial?**

-No, antibiotics. Antimalarial it might almost be the same. The same figure. Of course it depends where you are. When you are in OPD you prescribe more

**Share me what you know about factors that influence antimicrobial prescription? What I mean is what the patient factors that influences antimicrobial prescription are?**

-One could that most patients when they to the hospital like this they might have seen somewhere so they will tell, for example that they got antimalarial but did not improve. So from their story one might think that I think this time because they got antimalarial they did not improve. If they still presenting with the same, one would be compelled to let’s say let prescribe antibiotic. It could be the patients themselves forcing the clinicians that I feel like if I get certain antibiotic they might mention it, **Penicillin’s**. They normally say, could you prescribe me **Penicillin’s**, Pen V. Yeah, so it could be from patients. We cannot run away from clinicians themselves. Sometimes one would say we just want to please the patient and can prescribe the, yeah.

So you have mentioned three factors. Patients have seen the medication somewhere or they did get the medication but did not improve, patients may force clinicians to prescribe antimicrobials. They can mention penicillin, pen V. so you have also mentioned that pleasing the patient. Clinicians wants to please the patient.

**What do you think are the type of patients that clinicians want to please?**

-Mostly they might be friends or people they might be sent by somebody who is close to you. And you want to make sure, to look like you have assisted this patient when you prescribe more. Those are the most patients.

There is any other group that may request antibiotics or antimalarial?

-Well, those that look like they dress well, they have money or tend to over treat these patients

Okay. So you mention that sometimes clinicians want to please these kind patients especially those are your friends. You want to please them and prescribe them antimalarial. Those that have been sent by someone. You want to please your friends who have sent those type of patients. Those that are with money. Those that look like they are coming from wealthy family and have dressed well.

**So when did you start prescribing antimicrobial? Both antimalarial and antibiotic**

-Since 2007

You were already qualified that time?

-Yes, I qualified in 2007

So in 2007 you started prescribing both antimalarial and antibiotic?

-Yes

**What problems did you face during this period when you started prescribing antimicrobials?**

-The main problem I think we are still verifying in terms of information. Most our patients they don’t know information. They don’t know the problems if they keep on getting antimalarial, the problem that will come. The other problem is that most of the time clinicians we don’t have time to talk to our patients about treatment and the like. So those are the main issues that I, I mean main problems that I face. Of course sometimes the availability of antibiotics itself. You might think that this drug is worthy for this condition but if you don’t have it you are forced to prescribe another antibiotic that could work similar to what you had.

So you are saying that the problem that you face; lack of information especially to the patients, sometimes these patients do not know the problem that will rise when you prescribe this antibiotic. You also mentioned of time. You need a lot of time but there is large number of patients waiting for you. You also mentioned the availability of drugs. The drug that you want is not available you go the.

-What is recommended is not available you are forced…

**Okay. So can you explain to me your thought regarding patient factors and beliefs about antimicrobials?**

-I feel like, patients feel like whatever they get it will assist them and I have seen most of the patients coming to hospital they have in mind what they really want to get. For example they might come to hospital and say the way am feeling this is malaria. Just prescribe me antimalarial and I will be fine. Even if you test them **MRDTs** negative. On top of that what they think is what, they start getting medications. And when they start improving despite that the dosage is three days. But if they take a day, they feel better they think it’s better for them and they stop. So I think they are not really aware of the consequences.

So you have mentioned that they come and demand antibiotic depending on the way they present. They also demand antibiotic as they say this drug goes well with me, “I think I will improve”. You also mentioned that when they are better they have improved despite the dosage for seven days or three days they will stop there. They are feeling better. Without knowing the consequences that will rise. They will already have in mind the drug that they want when they are coming to us. They will say prescribe this drug it will be much better for me. Normally,

**What are the challenges do you encounter when prescribing antimicrobials?**

-So one of the challenges that we see is the same. Most of them I highlighted. It’s almost similar. You prescribe antibacterial and antimalarial, you expect your patient to take them but they don’t finish and mostly they might come back and well before a period of month you see them they are no feeling well because they didn’t finish the dosage.

And maybe another new problem is when you are working at an institution like this one you expect people to pay. Some will say I don’t have enough money to pay and they might go home without getting a full course and they end up buying. If they buy in pharmacies maybe they might buy dosage which is not full. I mean it wouldn’t be a dosage. Maybe they might if the course is for seven days they might, they will do it for two days and they will stop. So those are the challenges.

So you have mentioned not finishing the dose, if you have prescribed antibiotics they will leave at home which means someone will come and take them. Because this is a payment institution then they may not be able to pay so they will get a half r a quarter dose. When they go somewhere they will also buy another dose which is not full as well.

-Not necessarily getting a quarter dose because normally we here, we encourage them they should take a full course but they will say no I don’t have money, let me go home. They might because they have a prescription they can buy. Somewhere they can be, they can accept them to buy maybe two day course instead of seven days.

**In your view how do you describe the attitude of your patients when you refuse to prescribe antimicrobials? When you say No I will not prescribe antibiotics or antimalarial because of this. What does your patient do? Or how do they show to you?**

-In the first place, most of them will go home unhappy. But because I really give them information why I did that. When they coming next time for review because I normally tell them am not prescribing this medication because this will not help you. You don’t have that problem. What I will give you whether I say I will give **Analgesia** because he is feeling pain. Then I will give them a day to review. When they are coming back, they come back happy. If there is no improvement then I will keep on investigating to see what is causing that. But, initially when they are going they are not happy. And when they come back they realize the importance of that.

So they normally go unhappy

-Sure, they go unhappy.

**In your institution, suppose one of the patient has come to you to say the MRTD is negative but I take LA I feel better. In my entire life they have never detected malaria in me so when you refuse to that patient prescribing LA, what’s the attitude?**

-Still they are unhappy and they will try to find means. If you are so observant you will see them go to another clinician. So, institution like this one, most staff will come to a clinician who is so new. They know that they can manage to convince that clinician and prescribe them. So they will run away from those people who have stayed quite long.

So you are saying they will try to find another means. They will go to another clinician, convince him so that he should prescribe medication for them. So this type of patients do they come back to you?

-No they will not. You mean….

This kind of person when you have refused prescribing antimicrobials, do they come back or normally run away from you?

-I think they will normally run away. They will not come back. I think that’s why as an institution, initially we made a policy that for staff they need to be seen in the private and some people who are so**.** We did that deliberately because we knew that they will run away from and they will go somewhere. Because normally in the private there is always a senior person and the chief clinical officer is, the medical officer so those people can prescribe.

So you are saying that you have a staff clinic where all the staff are supposed to go there so that you monitoring**.**

**What communications skills are needed when you are prescribing antimicrobials?**

-Really you need to tell them what medication you are giving, why are you giving the medication, how long should they take. Of course these are expected to be told in the pharmacy but it doesn’t happen.so over the years I have noted that it is really good to discuss our patients their diagnosis, the management, how long should they take medication. The need not to share medication at home. If they are not improving they need to come back. And really you tell them the signs of worsening conditions. That will make them feel free to come back. The main problem with clinicians I think we don’t really develop rapport with the patient. We don’t have time to chat with them. And most of them, they take the hospital as a strange place. Most Malawians especially this setting, rural setting, they don’t feel comfortable in the hospitals. We need time really to chat with them. Of course I have a problem especially when you are in the Out Patient Department, where you have a lot of patients that you need to take care.

So you have mentioned quite number of things. You say why the medication should be given to them. Duration, they should know the duration. Should know what type of medication. They should also know the need to take that medication. They should also know how to come back

-And to add the consequences if they don’t finish the medication. It’s good to tell them.

You also added the consequences of not finishing the medication. They should not share the medication at home. There is also need of time for them to discuss what so ever is needed for that communication.

**So how much time do you spend with your patient?**

-It depends. There are some patients who are simple and straight forward. They will come to you vomiting, fevers, you examine them, nothing. You think of malaria. They test positive then it so doesn’t take time. Probably five minutes. But there are some patients who come in with complicate ones. It might take time. Those who want to admit for example. It might take you fifteen minutes, it might take you ten minutes but on average like in OPD for those with simple conditions we can have an average of five minutes.

So suppose the patient has come, the MRDT is negative, despite features of malaria. You have done the full blood count. All the parameters are normal.

On average what duration time can you spend with patient?

-MRTD negative? I think as I have already said five minutes should be enough for those patients. Unless maybe you are not convinced that it looks really malaria and you want to do other tests. For example, it’s possible I have seen people come MRDT negative and you send they come positive. Those that you want to check, to make sure that they don’t have malaria. And what you want to give is maybe just **Analgesia** and they go. You observe them. It might take another five minute or ten minutes.

**Could you describe some of the guidelines that are used during prescription of antimicrobials? Both antibiotics and antimalarial**

- Normally we are guided by the Malawi standard treatment guidelines. That for example if you thinking of malaria, what is the treatment. If it’s **Pneumonia**, what do you give, in consultation with the Malawi standards treatment guideline? Of course we use some other protocols that we have developed locally. We are guided. I think the main problem is not what to prescribe but the main problem is the diagnosis itself. Because one can put the diagnosis just to think the medications it’s giving. Like if you treating malaria for example. Somebody, the same patient can say this is severe **Pneumonia,** somebody can say this is just **Pneumonia,** let me give him **Amoxicillin.** Somebody will say no let me give antibiotics to this patient. The problem I see is probably how to make a diagnosis but in the terms of selection of antibiotics I think we are already guided, we have the guidelines.

Apart from MSTG, local protocols, any other?

-We have **BNF,**

Any other locally? Malawi local guidelines?

-Yeah, there are a lot. We have books for example, the college of medicine. We have books. We have the pediatric handbook for Malawi. **…** Kazembe and friend. We have a lot of manuals that we use. We normally have also journals that we read and can see.

In terms of other antimicrobials guidelines?

-Yeah, there are also TB guidelines.

Anything to do with the antiparasite guidelines?

- Yes, like we have the ART guidelines. I think there are a lot. We cannot say that we have problems with resources. We have internet access.

Are these guidelines locally available, placed in wards in our offices?

-Yes, they are locally available. We have even a library in the medical director’s office, where we can find these books in wards like labor wards and neonatal we have guidelines also pasted on the walls.

You mentioned of Malawi standard guidelines, TB guidelines, ART guidelines, protocols, BNF which is also used. The protocols, College of medicine, the pediatric handbook by Kazembe. The journal articles which have got also standards like the same that we are using. Have you ever heard of antimicrobial resistance?

-Yes

**In your own words, what is it bacterial resistant?**

-It is when for example you give medication to somebody and that somebody stops responding, I mean getting well to that. A good example would be, you are giving **amoxicillin** to a patient for **pneumonia** and is not responding to **pneumonia** because the organisms have been used to that antibiotic, I mean they have developed mechanism that they cannot, the antibiotic cannot work on those bacterial.

So you mentioned that this is just a resistance whereby the drug is not working on a particular microorganism because that microorganism has developed a mechanism for that drug not to work.

**In your mind, do you have any bacteria that have developed resistance?**

-Yeah, we know the micro-bacteria that is resisting to some of the TB medication. We know most bacteria that have developed resistance to **penicillins** and to specific mention the bacterias-we have I think **Shigera** which is now resistance to **Chloromphenial.** We know of the plasmodium resistant to **SP**, resistant to, am not sure about **LA** but I have heard that peoples say that there is some resistance as well.

Thank. So you have mentioned of TB resistance to some of the drugs. The **Shigera to Chloramphenicol**, you have mentioned the parasites, the plasmodium

-And even HIV is resistant, yeah

HIV as well resistant to some of the antiviral drugs.

**So in your own words can you describe what is meant by antimicrobial resistance?**

-Yeah, so it’s when whether its bacteria or viral **protozoa** it’s not responding to a drug it used to. Like previously you could treat with the same drug but now is not responding.

Thank you. So you have mentioned that antimicrobial resistance to these kind of drugs, whether its antiviral drugs, or antibacterial, **antiprotozoal.** These drugs were working to these microorganisms but they are no longer working.

**Could you please describe factors that could lead to antimicrobial resistance? Antibiotics and antimalarial, both.**

-The first one could be clinician, there are clinician factors and patient factors. Clinician factors, they might be carelessness prescription, there is no really indication for that drug to prescribe. A good example could be giving LA to somebody who has just fever with negative MRDT. It might be flu but you still giving LA.

The second one, the same prescribing to please somebody. And not giving still enough information, I would take it as a clinician factor. For those if they don’t finish their dose it means you haven’t given them, because of information there is a problem. Patients’ factor, then you will see that most of them do not finish their dose. Once they feel better they can stop. They share medications. They will force some clinicians to give them medication and if they can’t force them they will find their ways to buy. We’ve seen antibiotics in the market. Those are some of them, it might be I have forgotten some because there are really a lot. Adherence issues especially to some drugs like ART.

So you have mentioned that, clinician factors and patient. Clinicians, careless prescription. Prescribing drugs where there is no indications for example LA given to patients of **MRTD** negative. Clinicians wants to please someone, they will prescribe antimicrobials. Not enough information. Clinicians are still prescribing antimicrobials. On patient factors you have mentioned not finishing their medications, not finishing their doses. Patients will also share medication to other people. They can also go to buy in private areas when you have not prescribed them. Patients can also force clinicians to prescribe antimicrobials. You have also mentioned the issue of drug adherence to some of the antimicrobials like the **ART**. You have mentioned vomiting itself as a problem where by the patient is vomiting they stop taking medications. In terms of the same, on the antimicrobial resistance do you have any environment factors that can lead resistance?

-Infection prevention. If for example your environment is not clean you will expect people to get exposed to bacterial, viruses that could cause infection. Reinfection keeps on reinfection even if you have antimicrobials but the chances of resistance might be high. Health worker not following infection prevention standards for example examination, washing. Patients in Labour for example, wound care in the wards. They are a lot. Even in theatre we are not following proper hand wash procedure and the like. Preparation of the instruments.

On environmental issues you have mentioned issues of infections preventions, clinicians are not following up those standards that are supposed to use for example hand hygiene. In theatres, in the ward clinicians will just change from one patient to another without washing their hands, wound cleaning. In the wards. You also mentioned theatres. You have also mentioned instruments that can be used in patients, they can also transmit infections

-Another point is on isolation. Patients, those whose infectious conditions. Use of masks. Ventilation, our rooms.

**Whose responsibility is to resolve the problem?**

-I think it’s everyone’s responsibility. We need to work together; clinicians, nurses, patients, care givers. We need to work together. We need to give information. Maybe we could see how we can do this. Should we start in the OPD, everywhere patients can meet lets tell them about issues of antimicrobial resistance because it’s real. Maybe in every clinic we have keep to talking. Probably it will change. We should really empower patients to demand why are we giving? They should ask questions; why are you giving me this medications.

Every medication that clinicians prescribe they should really ask “why are you giving this? How would it work? “. If we do that, if we empower patients I think it will be good.

Thank you for attending this interview. That’s the end of our interview. Do you have any question or addition?

-No, I don’t have any question.

Thanks.
