# Supplementary material for: Determinants of clinician and patient to prescription of antimicrobials: Case of Mulanje, Southern Malawi
Source: PLOS Glob Public Health. 2022 Nov 16;2(11):e0001274. doi: 10.1371/journal.pgph.0001274 (PMC10022363; doi:10.1371/journal.pgph.0001274)
Supplement: S15 Text — (DOCX) [file pgph.0001274.s016.docx]

**15. APPENDIX:15, with Clinician number 15 on determinants of antimicrobial prescription at Mulanje District Malawi.**

I am Morris Chalusa. I am a clinical officer working for Mulanje hospital. I am also a student at college of medicine, doing masters of Science (Antimicrobial stewardship). As part of my academic, one of the recommendation is to do a research so I decided that I will do my research at Mulanje district hospital and Mulanje mission hospital. So I have also identified you as my participant. We will do our conversation in about 20 to 40 minutes. Questions that you see that are not appropriate to you, you are free not to answer them. If you want to stop the interview at any time you are free to tell me, we can stop. Our conversation will be kept secret and you are unable to find these interviews. You are also free not to mention your name, we’ll just start asking you question, thank you

**What is your role at this hospital?**

-I work as a clinical officer.

So what do you do every day in your work as a clinical officer?

-Currently I am the head of maternity department whereby I review patients and also I do some tutorials to the interns who are doing their attachments.

**Do you prescribe antimicrobials?**

-Yes

**Which one do you prescribe most?**

-Mostly, the antibiotics and also the antimalarial. Can I give examples of antibiotics that I normally give?

Yes

-The common antimicrobials that we normally give are **Ethromycine, Amoxilline, Benzylpenicillne, Ciprofracillixine and metronidazole** because we don’t give **prophylaxis** so we give Ceftriaxone but in terms when the patient has other infections we give according to the state of infection.

In terms of antibiotics do you combine both antibiotics and antimalarial or do you give each drug?

-No. We combine like giving one or at the same time?

Yeah

-Mostly I give them instantly.

In terms of antimalarial?

-The antimalarial, also it depends on the situation. Simple malaria and also complicated malaria and so we will treat accordingly. Simple malaria we will give LA, complicate malaria we give **Artersunate,** previously we use to give **Quinine** but currently we are giving **Artersunate**

**In average per day how many times do you prescribe antimicrobials?**

-Several times it will be more than three or four times.

**Would you share me patient factors that influences antimicrobial prescription? What do you think are the patient factors that would make you to prescribe antimicrobials?**

-Like to the patient themselves or for me to make the decision?

To the patient. Patient factors that would influence you to prescribe antimicrobials? Patient factors.

-Mostly it can be, of course I was looking at, am looking at it like suppose patient presents with I think patient presentation, examination and investigation. Those are the things that mostly I use to prescribe antimicrobials. So the presentation, the patient will tell me what she feels. And I have to examine to confirm whether patient has like I can give example of fever. Patient may say I have fever, then I have to examine and see if really the temperature is high. Then I have to investigate what’s the cause, then if the cause is known then I can give drugs.

Suppose the results of full blood count are normal, the MRDT are normal and what would be other factors that can influence you to prescribe antimicrobials? That would be coming from the patient.

-Some other times some other patients would just wish to have the drugs. They may try to, they may lie that we have this or they may still say despite everything is okay but they may still want drugs. So they can wish to have that.

So you have mentioned investigations, presentations as part of the factors that can influence you to prescribe antimicrobials. You have also mentioned patient wish, patient would lie. Sometimes they want you that you should prescribe the medication for them

-Yeah

**When did you start prescribing antimicrobials?**

-2011

**2011. Which ones do you prescribe most since that time?**

-Like all of them or I can just give example?

You can give example whether it’s antibiotics or antiviral

-Antibiotics which are the mostly commonly used are **Amoxicillin, Ethromycine, Gentamycin, Chloromphenial, Ceftriaxone, and also LA Artersunate** which are also malaria treatment.

**So since you have started prescribing antimicrobials what problems do you face during this period when you started prescribing antimicrobials?**

-Some other time you may give the patient drugs but patient may not improve on those drugs. Some other times you may find that possibly is resisting to the drug or the patient is not taking the drugs as instruct. There are some other patient who just take maybe the drug. If you give them for five days they just get two maybe three days and they feel are well they stop.

And they will come again with illness and you find out they didn’t finish the course of treatment that are there.

Any other challenge? You have mentioned about lack of improvement when you prescribe because of resistance, not finishing the prescribed dosage, when they feel better they will just take for two three days then they stop.

-Some other times I think the preferred drugs I can prescribe they are not available and that limits to just prescribe the drug which maybe possibly other drug I can give to the patient. Like most of the times **Azithromycin’s** are not common.

**There is any other problem that you face?**

-Of course there some other patients’ **myths** with drugs, they say I don’t take this because of. But the do give reasons which are not relating because they say I don’t take this drug because of the reason which if you have to look it, it’s not even a side effect that you can deny or you can opt to change the drug.

So you have mentioned about patient **myths**, not availability of drugs, challenges you started facing while prescribing antimicrobials. Can we go on?

-Yes

**Would you explain to me your thoughts about patient factors and beliefs regarding antimicrobials? What does your patient believe about antimicrobials?**

-Of course the beliefs and the myths comes with a new drug. For example when LA came in people thought that it’s a government system to make people not to be productive. So people were resisting to take it. And they just have a negative attitude with this drug like when I take LA I don’t feel better. So there are others issues that the patient may just believe which are not even the good factors that the drug is. Some other times I think it can be the patient they do have the drug that they believe most. They can just come from home thinking that when they come to the hospital I want to get maybe they want, (drugs that they want), so whatever you can prescribe but if they stick to that they will still push you to prescribe that medication without any reason for that drug. So those are the things, the factors that patient when they come in to influence me to prescribe. But I have to ware and counsel them and give them the appropriate drugs drug.

**Any belief from the patient?**

-Others do believe that other drugs are there to like I have already said they that the drug is meant to make them unproductive. And also they do believe that drugs that we give at the hospital there are there to, they are more of chemical which. They also believe that other diseases which are coming in like I heard one of the patient says I have a side effect, I think it may be due to the drugs. I have been using this drug so it’s the one which has caused that. But looking at the drug and its mode of action it cannot lead to that. It’s not one of its side effect.

**What challenges do you encounter when you prescribe antimicrobials?**

-The other challenges I think I have put them on that that the drugs are not available. I can prescribe drugs which are not available which I can evaluate you may see of it may be of help. Some other times I think the nurses are not willing to give drugs due to their thinking and also how they prefer it. You may prescribe, you have seen the child and decide maybe this child may benefit from **Artersunate**, going to the ward you find that patient is just put on LA due to the nurses. And also some other challenges can be the patients themselves. Because the patients before they come to the hospital they started I think buying drugs from another sellers. Then they take the drug for some days and if maybe they do stop at the end of the day if you prescribe the same medication they can take it home and not even take it. They can just take the medication and keep at home. According to me I believe that they are taking the drug whilst they didn’t take it. If they come maybe with the same presentation maybe they are not improving for me I would say they didn’t improve because the drug wasn’t working whilst they didn’t take it.

So on the challenges you have mentioned not availability of drugs, no available drugs, some others might not be available that you can give to the patient. Nurses not giving some other drugs because of beliefs. Some patients might already buy medication. And when they come here, you give them they go home not improving because you are giving them resisistant drug. Any more challenges? Or

-No, because currently I think those are the challenges that I face. Apart from that I think some other time of course we do have patients which are in rare cases which their beliefs or the churches which they don’t want them to take medications but in rare cases.

**Suppose you have got one of the workers here, supporting staffs. So they came to you, you see them and you have refused to give them antimicrobial, anything or antibiotic or antimalarial. What are the consequences? What will happen to that patient to you?**

-Of course in fact there are several times they have been denying people not to give them the drug they want. Of course there was not so much consequences apart from that they just have a negative attitude with me. But, I have to weigh if at all I give the drug like mostly antimalarial. I am one person who also coordinates malaria programme**.** If the patient is negative to the test is not supposed to get antimalarial but I think they may come to say I want since because ever since I was born I have never been positive of malaria so that cannot still provoke me or make me to give antimalarial or antimicrobials. I am supposed to weigh and investigate. So I the investigations say no, it’s not malaria I still not give. Yeah, of course they maybe anger with me but I have never faced any consequences because I have done that several times. But I have never had any consequences to say are talking a lot of things. They may say other things, they may have negative attitude but we do that.

**Do they still come to you?**

-Some they not but others they do come because I don’t just lie to them, I have to explain to them why am I doing that and why am not giving them that drug. And with the good counselling they do understand and if they go they get the medication which can assist them.

If they are not improving I tell them to come back. Not just giving them what they want.

**In your view how do you describe the attitude of your patients when you refuse to prescribe antimicrobials?**

-The approach I think maybe I used to tell them not to give them that drug. Out of ten patients maybe it’s only one which we can become angry that maybe they don’t want me to see them again but mostly I do counsel them and give them advice for them what they can take at that particular time. Out of those patients I have seen because I don’t just refuse I have to give them the reasons why am doing that. So with the reasons a lot of them do understand and appreciate the knowledge you have given to them.

**What communication skills are needed when you are prescribing antimicrobials to patients?**

-Like to my side, first I do tell them the diagnosis according to what you have presented, according the explanation and according to the investigations. You have got this condition so this is the medication am supposed to give you. So I will them how to take the medication, how many days, and when to come for review. So with all the explanation I give to the patient I think they do.

So in terms of communication you are saying tell them the diagnosis, duration of the medication, they take, what type of medication and review day. Anything on communication skill?

-I do also tell them the side effects of the drug. And they experiencing the side effects are supposed to come even before the appointed date they are supposed to come. And also advise**.**

Is there anything you can add?

-On communication I think because I think those are the basic things I do tell them. And also ask them if they understand. So feedback I think I have to receive back from them. They have a question they have to ask before they leave because when they get medication they should mix up things.

In communication you have mentioned diagnosis, duration of the drugs, review, which drugs are supposed to take, side effects advice on the, when on side effects when they should come back, and also they should understand. They should feedback when you are counselling them. So would ask them if they have understand the whole process.

-Yes, because initially the communication will start when the time I meet them, like I will introduce myself to them but I think after the investigations it’s when I go on and tell them but it starts when I meet them. I have to introduce myself to them. Ask them what their problem. Examine them. Ask them problem and after its when maybe I will, after the history, the physical examination I have to tell them what to be done, the investigations which I am intending to do. Then, they go there. After all the investigations and examinations I sum up with and come out with a diagnosis then communicate to them.

**How much time do you spend with each patient?**

-It depends how hard the situation but mostly I can spend ten to fifteen minutes but depending on the condition.

Because there is some other condition which are straight forward, the others which are complex. It depends so I can spend much time when the condition is complex. If I need to probe more. Of course we meet different patients. Others, they are skilled enough they can tell their history. Others they want you ask them questions. So depends.

So it depends with situation of the patient, I mean the disease of the patient.

-And even how skilled the patient explain his or her condition.

**And also skilled patient as well. I mean skill we are meaning knowledge?**

-Yeah the knowledge and also how to explain. Others they just say like an example to say I have headache, then you have to ask them is it frontal, is it temporal, is it. So others would say I have headache which is in frontal. They can explain everything before you even ask. So that lessens the time because the time you want to ask and respond they have already told what they are suffering.

**Can you describe some of the guidelines that you use when you are prescribing antimicrobials?**

-Mostly I do use Malawi standard guidelines which is available in my work station and also we do have the policies that we normally use as the hospital. So apart from that we also use the policy of the conditions that we meet and also we have the British formulae**,** the BNFs, and so there are some other books like I do have the college of medicine book, the blue book, I also extract some other information I want. So yeah I use those guidelines but apart from that we have the protocols which like in maternity we do have maternal protocols which tells you how to go about with the cases.

There is any other guidelines that you use?

-We do have the STI book and also the chance that we normally.

Have you ever heard of antibacterial resistance?

-Yes

**In your own words, what is it bacterial resistant?**

It’s the time where the person has taken the drug, you have prescribed the drug and maybe the patient has taken that drug according to the prescription but the patient is not responding. The condition is not changing but of course you have to do culture and sensitivity for you to prove that there was resistance.

**Do you have specific examples of bacterial resistant?**

-Like malaria I had few cases there resisting to **NOVIDA SP** which I saw that they were resisting. But according to the bacterial, they are both but no specific that we have done them because we don’t do **culture and sensitivity** and see which ones are resisting. So I don’t have a specific one but those cases do come.

**In your own words what is meant by antimicrobial resistance?**

-A situation whereby the patient is not or the bacterial are not eradicated to a prescribed medication or a certain antibiotic that you have given.

**What do you think are factors that will lead to antimicrobial resistance?**

-Some other times it can be patients themselves. They are not following or they are not taking the drugs according to prescribed. Here others they do take maybe today and skip maybe two three days and take the other day so that can lead to resistance. And also some other times it’s the unavailability of drugs which maybe you may have some few doses then a patient can get that and later on maybe two three days or two three weeks then the patient still takes the drug. You give them the same drug. So some other time out of stock of drugs can also lead to that.

**Any hospital related factors?**

-Yes some other times I think like in our case currently we don’t have the, we used to treat say severe **pneumonia** with **Benzylpenicillne and chloromphenial.** But now currently **chloromphenial** is not available. So suppose you start the patient on **chloromphenial** for the first maybe two three doses then going to the pharmacy you find that **chloromphenial** is not there. Now we need to change drug. So the hospital needs to be consistent in buying the drugs say if we have the drug, this drug in stock has to be readily available.

**Whose responsibilities is it to solve this problem of antimicrobial resistance issue?**

-Of course it’s both, us clinicians, the administrations and so even the patients themselves because you can give the drug but you will not be there to monitor when taking. They are home so they can choose when to take it and when not to take so it’s both, the patients and even the clinicians we need to give them knowledge. For some other time others, they do that out of ignorance. They don’t know so they need to be given information so that when they are doing that they have to have knowledge to say I have to do this. At the end of the day maybe resist to this drug.

**Do you have any additions?**

-No

So that’s the end of our interview. Thank you for participating

-Thank you so much
