# Supplementary material for: Determinants of clinician and patient to prescription of antimicrobials: Case of Mulanje, Southern Malawi
Source: PLOS Glob Public Health. 2022 Nov 16;2(11):e0001274. doi: 10.1371/journal.pgph.0001274 (PMC10022363; doi:10.1371/journal.pgph.0001274)
Supplement: S16 Text — (DOCX) [file pgph.0001274.s017.docx]

**16.APPENDIX:16, focus group discussion with six medical assistant, on determinant of antimicrobial prescription at Mulanje District Hospital, Malawi.**

**INTERVIEW, FOCUS GROUP DISCUSION**

‘I’m Morris Chalusa, a student from the College of medicine, University of Malawi, I’m doing Master of Science in Health Sciences and antimicrobial stewardship. I’m doing a study called DETERMINANTS OF DECISIONS BETWEEN CLINICIANS AND PATIENTS TO PRESCRIBE ANTIMICROBIAL: A CLINICIAN PERSPECTIVE. It’s a quantitative study actually. We will have focus group discussion that will take as to 30 minutes up to one hour. I have a guide for your interview so in this interview, you are free to participate, you are also free to withdraw anytime that you feel you have been offended, you are also free to not answer any questions that you feel are not relevant to you or they are irritable and you are also free to not mention your name in this interview. The recording will be kept safe and the only person who will be able to access this recording is myself, the principal investigator, my supervisor, and the one who is going to help me in analyzing.

**What is you role you’re at the district hospital**

Intern medical assistant

Intern medical assistant

Qualified medical assistant

Qualified medical assistant

Qualified medical assistant

**How long have been at this district hospital**

Have here for twelve months and in Pediatrics wards

7 month and in peadiatric ward

1 and 2 months and in under five

5 years and am in under five

7 years and am in under five

**Do you prescribe antimicrobials both antimalarial and antibiotics?**

Mostly antimalarial

Mostly antibiotics

Mostly antibiotics

Both antimalarial and antibiotics

Both antimalarial and antibiotics

**Why do you think you do prescribed what you have mentioned?**

I prescribed antimalarial mostly of the cases come are for Malaria

Most drugs are that i prescribed are antibiotics because most cases are supposed to be treated with antibiotic such as cotrimocozales that is there in our pharmacy.

I prescribed antibiotics because most of the cases are cough

It is almost the same, when patient is presenting with Malaria we prescribe antimalarial and if present caught we prescribe antibiotics

It is the same because there are common condition

**On Average per how time do you prescribed antimicrobial both antibiotic and antimalarial?**

One hundred to one hundred fifty patients

I depend with number of patient seen per days

Depends on the days like Monday the many patients

**‘Will you share with me the patient factors that influence antimicrobial precribition?’**

When someone has diagnosed to have malaria you can prescribed antimalarial

When some have been diagnosed to have malaria and pneumonia you can prescribe antimalarial and antibiotics

In casualty department and patients has involve road traffic accident and the impact is big you ca prescribed ceftriaxone cover that injury

Patient comes with signs of Malaria and MRDTs is positive you prescribed antimalarial.

When patient presenting with persistent fevers and MRDTS is negative do prescribed antibiotics

SP can also be prescribed in pregnancy woman as a prophylaxis

We prescribed when we have asked from patients,

Sometimes is challenge when you coworkers has asked to prescribed

**Supposed you have been asked by a core worker to prescribed, are not going to prescribed antimicrobial?**

The is not good to prescribed antimicrobial in the absence of patient even if core has asked you to prescribe you to tell the core worker to bring the drug, it is good to let someone come to the hospital so that you and examine the patient for proper treatment.

Suppose Malaria diagnostic test is positive and patient is coming from the ward not the and there is no evidence are going to prescribe antimalarial

Yes we will prescribe antimalarial, but the best option is to repeat the test for Malaria Diagnostic test at the OPD. Present and history of patient symptoms you can also prescribe antimalarial despite being negative. Sometimes you can tempted to prescribe antimicrobials and antimalarial when someone comes with and said I will give you so so amount of money.

**When did you start prescribing antimicrobials?**

2012

2014

2016

2017

2017

**What problems do face when you started prescribed antimicrobials is you stared prescribing?**

Guardian will come that drug you gave me my child is not improving this are challenges that we face that because you never know that bringing to improve with LA antimalarial. Sometimes guardians will say that my when take this medicine does not work I want quinine, I don’t want cotrimocozales and Amoxiciniline, they will tell that drugs or not working but you more thoroughly exams there is poor drug compliance, other challenges unavailability of drugs some drugs are not available such as cotrimocozales only, some will come asked to be prescribes specific drugs. Previous visit patient come and was given medication by clinicians when he or she come to second for next visit will asked I want this drugs that I was given before. Some will come with treatment failure like you have prescribe LA and it is not working.

**What are patients’ belief about antimicrobials both antibiotics and antimalarial?**

They think that cotrimocoxazole that it is not effective and it does work, it is cheap,

In terms of antimalarial when they presented with fever, headache and chill they this are symptom of Malaria and if malaria diagnostic is negative and you give them cotrimocoxazole they don’t feel good.

Some will say that cotrimocozales will give them skin rashes that what they believe, some believe that LA will not help them but quinine and they will ask you to prescribe quinine. They believe that any cough is equal to antibiotics.

**How do you describe attitude of your patients when refuse to prescribed antimicrobials?**

Mostly if don’t prescribe antibiotics and malaria they that you don’t know what you’re doing and dull, and they feel that you greedy one and negative attitude with you because you did not prescribed they want, if your counsel them they agreed, they also angry at you.

**What communications skill are needed when you’re prescribing antimicrobials both antibiotics and antibiotics?**

It is matter of convincing the patient that you come with this and that and after carried investigations and guideline you should take this medicine and provide good communication skills with what is presenting, you have to tell them that this drug will be taken twice of three time days, you have also to kame good interface between you and the patient so that is convinced what you are telling.

**How much time do you spend with each patient?**

3 to 5 minutes

It depend with the department that you are working like STI clinic to can reach up to ten minutes.

It depends with the condition patient presentation.

It depend with condition and place where you working like where there is no privacy it means you spend less time.

**What are the guidelines that are used when you’re prescribing antimicrobials by clinician both antibiotic and antimalarial?**

Malawi standard treatment guideline, peadiatric book Kazembe, Clinical Book clinical, malaria Guidelines STI Guideline HIV and AIDs guidelines and Google

**What is Bacterial resistant?**

When bacterial was cure by a certain drug and that now is not cure currently it is resistant from that drug

**Give Antibiotics that are resistant to bacterial?**

Cotricomoczaxole, amoxicillin, Benzylpenicillne, ciproflaxilline, tetracycline, metronidazole.

**What is meant by antimicrobial resistant?**

It is when antibiotics and antimalarial given to patients are no longer working

**Factors that will antimicrobial resistant both antibiotics and antimalarial?**

Frequent taking of drugs

Drug compliance

Overprescribing

Taking drugs without prescription

Taking drugs without having a disease

Taking expire drugs

Not finishing the required drugs

**Who’s responsible to solve this problem?**

Prescribes should prescribe recommended dose and they should take them

Outcome should depend from the prescribed and patients as prescribed if you’re going to emphasis the dosages and time of medication we might end up with resistant.

Miss understanding between the prescribes and patients, what happens they cannot drug and can buy drugs at a private clinics.

**Thank you a ladies and gentlemen for participating in the study**
