# Supplementary material for: Determinants of clinician and patient to prescription of antimicrobials: Case of Mulanje, Southern Malawi
Source: PLOS Glob Public Health. 2022 Nov 16;2(11):e0001274. doi: 10.1371/journal.pgph.0001274 (PMC10022363; doi:10.1371/journal.pgph.0001274)
Supplement: S17 Text — (DOCX) [file pgph.0001274.s018.docx]

**17. APPENDIX:17 on focus group discussion with clinical officers on determinants of antimicrobial prescription at Mulanje Mission Hospital, Malawi.**

**INTERVIEW, FOCUS GROUP DISCUSIONS**

‘I’m Morris Chalusa, a student from the College of medicine, University of Malawi, I’m doing Master of Science in Health Sciences and antimicrobial stewardship. I’m doing a study called DETERMINANTS OF DECISIONS BETWEEN CLINICIANS AND PATIENTS TO PRESCRIBE ANTIMICROBIAL: A CLINICIAN PERSPECTIVE. It’s a quantitative study actually. We will have focus group discussion that will take as to 30 to 60 minutes up to one hour. I have a guide for your interview so in this interview, you are free to participate, you are also free to withdraw anytime that you feel you have been offended, you are also free to not answer any questions that you feel are not relevant to you or they are irritable and you are also free to not mention your name in this interview. The recording will be kept safe and the only person who will be able to access this recording is myself, the principal investigator, my supervisor, and the one who is going to help me in analyzing

**What is you role at this hospital?**

Clinical officer and my role is assess patients and prescribed antimicrobials

Clinical officer assessing and prescribing antimicrobials

Clinical officer my is the same apply

Clinical officer the same

Clinical officer the same as well and assess patients based on their complaints

**Where do you conduct majority of your work?**

Majority of work is in the wards and sometimes outpatient department

I work in female ward and outpatient department

Mainly in the paedriacs wards and casualty

I work in Antenatal, Labour and postnatal

I work in Antenatal, Labour and postnatal

Outpatients department

**Do you prescribe antimicrobials both antibiotics and antimalarial?**

Mostly this seasons is antimalarial

Antimalarial and antibiotics

Depending on the presentation you can prescribed both

It is both depending on the presentations

In peadiatric is the same both antibiotics and antimalarial

It is the same as my friends said because sometimes patients can need both

**In average per day how many time do you prescribe antimicrobials both antibiotics and antimalarial?**

Antimalarial 70 % and antibiotics 50 % in OPD

Maternity much is antibiotics than antimalarial we don’t reach up to 50% of those that get antibiotics and we don’t reach 10 % of those that get antimalarial.

In pediatrics is almost

In female ward 80 % of antibiotics and 20 % of antimalarial

**Share with me what patients’ factors influence you to prescribe antimicrobial?**

Like patients that present with fever and we have made a diagnosis of sepsis and we have ruled out other things we cornered to prescribe antibiotics depending on the examination that was done. In peadiatric ward we do prescribe more of antimalarial because children and neonate Malaria are more common. In Labour depend on assessment and what you found, for example if your have woman who have come with wound infection on the Caesarean section and fistulae we do give antibiotics while we are continuing with investigation.

Some patients will come with their own diagnosis and demand that if you give LA does help but quinine, secondly sometimes is what they are presenting if they are with Malaria or Bacterial infection is the one that tell you what to prescribe. Sometimes the can come with Viral infections but if you don’t give them antibiotics they don’t feel like you have help them, they insist and if you don’t give them they come the following days the keep come, keep coming. Sometime when you tell them that it is Viral infection and you give them paracetamol and aspirin, the will exaggerate and come with different clues so that they should convince you to prescribe antibiotics. Sometime the person might be senior to you so asked you I want this drug and sometime they are well to do and they may influence to prescribe antimicrobials. Sometimes patients will come with general body pains and you Malaria diagnostic test you found is negative, parents will tell you my son have been found with malaria but when he take LA he will be fine and you cornered to prescribed it. Sometimes if he is your friend will come and tell you that I want amoxicillin then you just prescribe because of friendship. Sometimes patients will last time you gave this one and can you prescribe me the same medication even if you tried to insist and explain that condition are different.

**When did you start prescribing antimicrobials?**

My I started when I was in school under supervision and after qualified now I stared prescribing on my own now it ten years. Since 2014 I was prescribing under supervision when I was at school, there years ago I was prescribing undersupervsion and two years later after staring internship and I was prescribing with limited internship and now am prescribing alone, in my case it is years now prescribing without supervision after qualified, four years now without supervision.

**What problems do you face when you stared prescribing antimicrobials both antibiotics and antimalarial?**

When you prescribe antibiotics we know the guidelines especially the first line patients have they own favorite said I want this type of drug this one not even if you try to reason with them and with the issue of so many clinics they will go to private clinics to buy antibiotics they want, unavailability of antimicrobials especially broad-spectrum are not available in health facility so it’s a kind of challenge. I even went to a private clinics and I saw that clinicians prescribe upper level antibiotics lie injectable so that they should make money, so it is challenge to see are prescribing antibiotics in order to make money. Sometimes you see a child and symptoms of infection and you start antibiotics whiling you’re waiting for investigation and lab test come normal and you stop antibiotic it’s a challenges. Sometimes you see a patient has come and see in health passport book and you found that patient has been visiting different hospitals and has already taken different antibiotics look for antibiotics and then your cornered you go to a different one. Unavailability of strong antibiotics is also a challenge. Another challenges is patient is patient can buy medication in drugs stores, pharmacy, and clinics and patients they don’t finish there antibiotics when you prescribed them they wait some other time when they are to take medications this is also a challenges.

**What does your patient belief about antimicrobials both antibiotics and antimalarial?**

Some belief that they work and belief that they don’t work and some has they own references. The main belief is preference because current we are using LA but others will still going for SP. They belief that if you give them antibiotics they will see dramatically change within two days if they have fever they want it go away within two days and if they don’t improved they come back. Some parents belief that when they child have got cough they belief that amoxicillin will work they don’t know that it can be cause by viral infections, and when child is present with fever and headache they believe that is Malaria and they will demand antimalarial even if MRDTS is negative. Most patient belief that IM and IV antibiotics works better than PO so you give the Po antibiotics they belief that you have help them.

**Supposed you are the outpatient department and you see patient you do full blood count and Malaria diagnostic and results are normal what is you challenge in the management of this case?**

Challenge is to provide counselling to such kind of case even if provide main killer patient will not get satisfied patient believe that something is going wrong they body. For you to convince that sick but they sickness does not need antibiotics or antimalarial is very big challenge, will not go home without medication, what they will do they go to the next door where is another clinicians or they go to private clinics or pharmacy where they can buy drugs. Client also does not believe that counselling is management of certain conditions but if don’t counsel them they will go to private clinics where they can get medications. Yes is true that when Malaria diagnostic test is negative and Full Blood cunt is normal you can give antibiotics and antimalarial but the challenge come when patients is sick and you cannot just give pain killers, it’s like that clinicians you don’t know what you’re doing.

**Describe the attitude of patient when you refuse to prescribe antimicrobials both antibiotics and antimalarial?**

They feel like you haven’t help them, they feel like you don’t what you are doing and they should go find someone else who can help them, they feel like they have underrated by the symptoms as clinicians we are supposed to counsel them and tell them if symptoms do not go away. They do come over the night since you did not help them. They feel like you have not assisted them.

**What are communications skills are needed when you are prescribing antimicrobials to patients?**

First they should know what they are suffering from, apart from that they should understand the other conditions that present the same if don’t give antibiotics. It also good to explain what is the diagnosis and how they are going to take medications sometimes will leave on the hand of pharmacy between to explain to them, know side effect and frequency,

**How much time do spend with each patients?**

It depends on the queue outside, some 2 minutes and 15 minutes and condition of the patients, like malaria diagnosis is positive it means less minutes, sometimes understanding of the patients also help you how much time can you spend with the patient.

**The time that you have mentioned 2 minutes and 15 minutes how does it affects your relationship with the patients, how you prescribe antimicrobials?**

Like patient has come and is saying that am having headache before he or she finished you have already written Malaria diagnostic, you need to give them a chance to explain. Few minutes you miss a lot of things, because you don’t probe more. If you said patients come early in the morning I should asked few minutes they feel happy but you are missing a lot of things and diagnoses. So patient doctor relationship depend how much you spend with them, if your too fast they think you’re not helping them so a good rapport is good with your patients.

**Describe some of guidelines used when you’re prescribing antimicrobials both antibiotics and antimalarial?**

Sexually Transmitted Infection, antimalarial, ART, TB, Antimicrobial.

**Have ever heard of bacterial resistant and what is it?**

It when an antibiotics used to treat a certain condition but now is not working on that condition

Examples of bacterial resistant,

Amoxilline, Cotrimoxazole, ceftriaxone, chloromphenial, gentamycin.

**What is meant by antimicrobial resistant?**

It is when a certain type of drug that used to treat specials of bacteria, special of parasites is longer working

**Describe factors that leads to antimicrobial resistance?**

Ignorance part of the patients, there are other that sell drug, if child is sick they will go to buy a drug that they don’t even finish the does, unnecessary and unnecessary taking of drug. Not taking full dose of antibiotics which results in under dose, more antibiotics are commonly found on the marked, share of medications among patients if they feel like they have the same symptoms at the community, long frequency of medications can also contribute also .fear in health facilities where there is no equipment for investigations so clinicians will say this is an infection then they do prescribe medications. Shortage of antimicrobials where by in health center there are few clinicians will opted to prescribe few doses so that they can prescribe to large number of people.

**Whose is responsibility to resolve this problem and why?**

Both, prescribers and patients, clinicians because our role is to do full assessment, talk good history, find the real problem and explain to them patient every nececceary thing that they have to learnt on antimicrobial resistant for them to understand.

To the patient, it is challenge because there are people out there who sell the drugs who does know how antimicrobials work like some selling two dose of antimicrobials, so we should explain them, role and how antimicrobials work and how take them and they should to specific clinics. In private clinics they just prescribe anyhow in order to make money. Policy make should make sure antibiotic should not be sold anyhow. So emphasis should be spread to patients that this drug are not working and there or resistant’s.
