# Supplementary material for: Determinants of clinician and patient to prescription of antimicrobials: Case of Mulanje, Southern Malawi
Source: PLOS Glob Public Health. 2022 Nov 16;2(11):e0001274. doi: 10.1371/journal.pgph.0001274 (PMC10022363; doi:10.1371/journal.pgph.0001274)
Supplement: Attachment — Submitted filename: Response to Reviewers.docx [file pgph.0001274.s019.docx]

C/O Mulanje District Hospital

P.O. Box 227.

Mulanje

Dear Editor

**Major corrections on manuscript Title: Determinants of Clinician and Patient to prescription of antimicrobials: case of Mulanje, Southern Malawi.**

I thank the reviewers for generous comments on my manuscript and I have edited the manuscript to address the concerns. The following are comments from reviewers that I have made.

1. Detailed online Financial Disclosure statement.
2. Funding Information.
3. Update your online Competing Interests statement.
4. Data will be uploaded as supplementary information.
5. The title in the manuscript and the title on the online submission form are the same
6. The figure found on page 7 has been removed because it does not affect the manuscript.
7. A full list of legends on the supporting Information files has been made.
8. Review Comments to the Author comments #5, changes have bee made with track changes. This includes line numbers that were recommended for corrections changes and additions.
9. Reviewer # 1and #2: Corrections have been made as recommended with editing as well, see the manuscript with track changes and without track changes.

Waiting for your quick reply

Kind regards

Morris Chalusa
