# Supplementary material for: Determinants of clinician and patient to prescription of antimicrobials: Case of Mulanje, Southern Malawi
Source: PLOS Glob Public Health. 2022 Nov 16;2(11):e0001274. doi: 10.1371/journal.pgph.0001274 (PMC10022363; doi:10.1371/journal.pgph.0001274)
Supplement: Attachment — Submitted filename: Response to Reviewers.docx [file pgph.0001274.s020.docx]

C/O Mulanje District Hospital

P.O. Box 227.

Mulanje

12^th^ October, 2022

Dear PLOS ONE Editor

**Major corrections on manuscript Title: Determinants of Clinician and Patient to prescription of antimicrobials: case of Mulanje, Southern Malawi.**

I thank the reviewers for generous comments on my manuscript and I have edited the manuscript to address the concerns. The following are comments from reviewers that I have made.

1.Reviewer # 3, on line 121correction has been made.

2. Reviewer # 3, (a) Are microbiological analyses (antigen tests, culture, PCR, or other tests) available to the clinicians? Are they used? We do not do microbiological analyses (antigen tests, culture, PCR, or other tests) for bacterial infections at the district hospital in Malawi, only antigen test for Malaria and HIV are done.

(b) Are there national or regional guidelines for empirical antimicrobial treatment? Guidelines for treatment when the causative agent is known? If these exist, are these guidelines known to clinicians? Yes, but clinician do not follow them due to factors like we found in the study.

3. Was data collection finished before or after the transcripts were read and analyzed? Answer, transcripts were analyzed after data collection.

4. Currently we do not have data on the experience of clinician who took part in the study, during the study time experienced clinician were not available because of other things going for upgrading in school.

5. Did you notice any differences in the themes expressed depending on if the clinician was a

medical assistant, clinical technicians, or clinical officer, or were the themes universal? No they were no difference in in terms of answer.

6. Definition used for "clinician" in the context of the 70 clinicians? Line 58 and 60 include the definition. While the proportion of medical doctors, clinical technicians is 3:60 at Mulanje district hospital.

7. In-depth interviews and focus group discussion had similar answer, one focus group discussion was for medical assistant with 6 participants while the other was for clinical technicians and clinical officer with 6 participants.

8. correction has been made lines 194-196.

9. On lines 202-203 correction made, it is stated that patients go to another clinician if antibiotics are not prescribed. Was this a belief of the study participants, yes,

10. On lines 344-345, in the discussion, it is stated that in the current study, clinicians did not

perceive limited time to lead to inappropriate prescription of antimicrobials, this sentence has been deleted to avoid contradiction.

Reviewer # 4

1.To me sound good because clinical officer, medical assistance are all called clinicians in Malawian health care settings.

2. medical assistants, clinical technicians and clinical officers added in the abstract.

3. correction made in methods part of the abstract.

4. Correction made in conclusion on line 386 -387.

5. Interventions should target both clinicians and patients, now in our settings, it has been proven that intervention that start from primary, secondary schools works rather than starting with already matured people.

6. On the key words “patients” has been remove.

7. Overprescribing of antimicrobials is associated with increased risks of prolonged hospital stay, self-medication of self-limiting conditions, amplified frequent admission to hospital and causing severe infections, this means overprescribing will contributed to left over medicine in which patients do not take antimicrobials and that will read to self-medication.

8. Correction on reference [3] has been made.

9. Correction on Reference [5],[6].

10. lines 50-54 deleted

11. Reference [8] - correction has been made

12. Reference [10] quantify in terms of proportion of global use rather than using words like enormous. actually in 2018 Who develop platform for collecting this, I try to search but no results yet. Since 2017, WHO has piloted the WHO Protocol for Point Prevalence Survey (PPS) on Antibiotic Use in hospitals in several countries.

13. correction made on the introduction.

14. In methods how was inappropriate antimicrobial use defined.? using antibiotics unnecessarily (no clinical indication for antibiotics) or not using antibiotics when needed according to clinical guidelines.

15. correction done and define the boundary that this qualitative study looks at done. On line 117.

16. corrections made strength and limitation of the study.

17. I have attached code sheet of themes.

18.Table correction made and this table was developed based on participants attendance.

19. Would be interesting to know whether COs admitted poor training in Antibiotic prescription.?. Was this probed.? This was not probes from the clinicians.

20. Multiple sentences have been corrected.

Waiting for your quick reply

Kind regards

Morris Chalusa
